# Midkine‐Mediated Microglia Activation after Renal Injury Promotes Cognitive Impairment Following Ischemic Renal Injury

**DOI:** 10.1002/advs.202507832

**Published:** 2025-11-23

**Authors:** Li Lu, Bixiao Liu, Yu Yang, Tao Meng, Yuanyuan Chang, Yao Peng, Jia Guo, Zhuqing Wang, Hongqian Guo, Liuhua Zhou, Xiaozhi Zhao

**Affiliations:** ^1^ Department of Andrology Nanjing Drum Tower Hospital the Affiliated hospital of Nanjing University Medical School Nanjing Jiangsu 210008 China; ^2^ Department of Urology Nanjing Drum Tower Hospital The Affiliated Hospital of Nanjing University Medical School Nanjing Jiangsu 210008 China; ^3^ Department of Andrology Nanjing Drum Tower Hospital The Affiliated Hospital of Nanjing University of Chinese Medicine Nanjing Jiangsu 210008 China

**Keywords:** acute kidney injury cognitive impairment, microglia activation, midkine, P2ry12

## Abstract

Acute kidney injury (AKI) is associated with a high prevalence of cognitive impairment, the underlying mechanisms remain elusive. This study explores the role of midkine (MDK), upregulated in renal injury, in mediating cognitive dysfunction following post‐ischemic renal injury. Using a mouse model of unilateral renal ischemia‐reperfusion injury, cognitive deficits and blood‐brain barrier disruption is observed. Single‐cell RNA sequencing and ligand‐receptor interaction analysis reveals a strengthened MDK‐LRP1 axis in both the kidneys and hippocampus of mice subjected to ischemic renal injury. MDK, mainly from injured renal tubular cells and fibroblasts, is enriched in peripheral blood and the hippocampus, correlating with increased activation of hippocampal microglia and upregulation of c. It is demonstrated that MDK internalization into microglia via LRP1 upregulated P2ry12 expression, promoting microglial activation and phagocytosis. Inhibiting renal MDK expression with shRNA adenovirus ameliorated cognitive dysfunction and attenuated microglial activation after ischemic renal injury. These findings suggest the MDK‐LRP1 pathway is a key mediator of cognitive dysfunction following ischemic renal injury and a potential therapeutic target for mitigating cognitive decline in AKI patients. It provides a mechanistic link between renal injury, neuroinflammation, and cognitive deficits, highlighting the potential of targeting MDK‐LRP1 signaling to address cognitive impairment after ischemic renal injury.

## Graphical abstract

The mechanism of secondary cognitive impairment following AKI. When renal ischemic injury progresses to fibrosis, renal fibroblasts and damaged tubular cells secrete MDK, which circulates through the bloodstream, crosses the damaged BBB, and accumulates in the hippocampus tissue (an area crucial for learning and memory). The Morris water maze test is used to evaluate the cognitive function of mice subjected to post‐ischemic renal injury, and the results show that AKI mice exhibits cognitive dysfunction, which may be the result of renal fibrosis and its impact on brain function. In hippocampal tissue, MDK is internalized into microglia through the Lrp1 receptor, triggering an inflammatory response in microglia, releasing inflammatory factors, activating the expression of P2ry12, and activating its phagocytic function. The functional changes of microglia mentioned above are considered as a factor leading to cognitive dysfunction. Overall, this study emphasizes the complex interplay between kidney health and cognitive function, indicating that AKI may have profound impacts beyond the kidneys, affecting brain health and cognitive abilities.

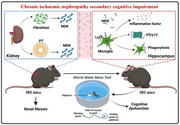

## 1 Introduction

Acute kidney injury (AKI) is a severe clinical syndrome with an incidence of ≈13.3% among hospitalized patients globally, leading to significant morbidity and mortality.^[^ ^1^ ^]^ The progression from AKI to progressive chronic kidney disease (CKD) is associated with a substantial health burden, affecting an estimated 10% of the global population, and frequently accompanied by various complications, such as cardiovascular disease and metabolic disorders.^[^ ^2^, ^3^ ^]^ Notably, AKI patients are at an increased risk of developing cognitive impairments, with prevalence rates ranging from 30% to 60%.^[^ ^4^ ^]^ The severity of cognitive dysfunction closely correlates with the stage post‐ischemic renal injury, cognitive deficits become more pronounced as renal function deteriorates.^[^ ^5^, ^6^, ^7^, ^8^ ^]^ Importantly, cognitive impairment may also negatively impact AKI progression by influencing patient adherence to treatment plans and overall self‐management of the disease.^[^ ^9^ ^]^ Therefore, early identification and intervention are crucial for slowing cognitive decline and improving patient outcomes and quality of life.

Although the accumulation of uremic toxins is widely recognized as a central mechanism underlying cognitive dysfunction among patients with AKI, it is not the sole contributor.^[^ ^10^ ^]^ Traditional cardiovascular risk factors, such as hypertension, diabetes, and hyperlipidemia, significantly contribute to cognitive impairment after ischemic renal injury.^[^ ^11^ ^]^ These multiple factors interact in complex ways, increasing the complexity of cognitive dysfunction. Emerging evidence suggests that injured kidneys may produce and release various proteins or metabolites that are transported via the circulation to distant organs, contributing to their dysfunction.^[^ ^12^ ^]^ For example, the release of inflammatory cytokines and chemokines from the injured kidney in AKI, can lead to increased vascular permeability and disruption of the blood‐brain barrier (BBB), resulting in neuroinflammation and cognitive deficits.^[^ ^5^, ^13^, ^14^ ^]^ Similarly, exosomes released from damaged kidneys in AKI models have been shown to contain elevated levels of inflammatory cytokine mRNA, which may participate in organ‐to‐organ communication and contribute to distant organ damage.^[^ ^15^, ^16^ ^]^ However, the specific factors produced by injured kidneys in CKD that directly impact cognitive function remain to be fully elucidated. Consequently, there is a pressing need for further research into post‐ischemic renal injury‐related cognitive impairment.

Midkine (MDK) is a highly conserved heparin‐binding cytokine.^[^ ^17^ ^]^ In healthy adult kidneys its expression is low to undetectable, but it is markedly up‐regulated following ischemic or toxic injury.^[^ ^18^ ^]^ Elevated serum and urinary MDK levels have been observed in advanced AKI/CKD patients, suggesting its potential as a biomarker for renal injury.^[^ ^18^ ^]^ MDK, as a multifunctional cytokine, is also involved in the development of various central nervous system diseases.^[^ ^19^ ^]^ Studies have shown that abnormal upregulation of MDK has been detected in the serum of patients with different central nervous system diseases characterized by neuroinflammation.^[^ ^20^ ^]^ In addition, the absence of MDK can reduce neuroinflammation, neuronal apoptosis, and alleviate memory deficits.^[^ ^19^ ^]^ These studies strongly suggest that the abnormal upregulation of MDK may be closely related to the development of kidney injury and the occurrence of cognitive dysfunction. However, it remains unclear whether MDK drives secondary cognitive impairment after ischemic renal injury.

Microglia, the resident immune cells of the brain, are integral to the brain's immune system and play a pivotal role in modulating higher cognitive functions.^[^ ^16^, ^21^ ^]^ Under normal conditions, microglia maintain brain homeostasis by clearing cellular debris and regulating synaptic plasticity.^[^ ^22^ ^]^ However, under neuropathological conditions, these cells can swiftly transition into activated states, exhibiting enhanced phagocytic capabilities and releasing pro‐inflammatory cytokines.^[^ ^23^, ^24^, ^25^, ^26^ ^]^ However, the abnormal activation of microglia can precipitate excessive synaptic pruning, a process by which unnecessary synapses are eliminated, can lead to synaptic loss and consequently trigger cognitive impairments.^[^ ^27^ ^]^ In the context of AKI/CKD, the systemic inflammation and accumulation of uremic toxins can lead to the activation of microglia.^[^ ^28^ ^]^ This activation may disrupt the delicate balance of synaptic plasticity, resulting in cognitive deficits.^[^ ^29^, ^30^ ^]^ The intricate crosstalk between renal dysfunction and microglial activation underscores the need to elucidate their underlying mechanisms and develop effective therapies for AKI‐associated cognitive impairments.

This study aims to elucidated the pathogenesis of cognitive disorders following post‐ischemic renal injury, particularly the role of MDK in cognitive dysfunction following renal injury, and the function of low‐density lipoprotein receptor‐related protein 1 (LRP1) in mediating the internalization of MDK and the activation of microglia. We propose the hypothesis that MDK secreted by the injured kidney binds to LRP1 on microglia in the hippocampus, activating the phagocytosis of microglia, which leads to cognitive dysfunction. This study may offer novel therapeutic strategies for managing cognitive disorders associated with post‐ischemic renal injury.

## 2 Results

### 2.1 Cognitive Impairment and BBB Disruption after Post‐Ischemic Renal Injury in Mice

**Figure 1:**
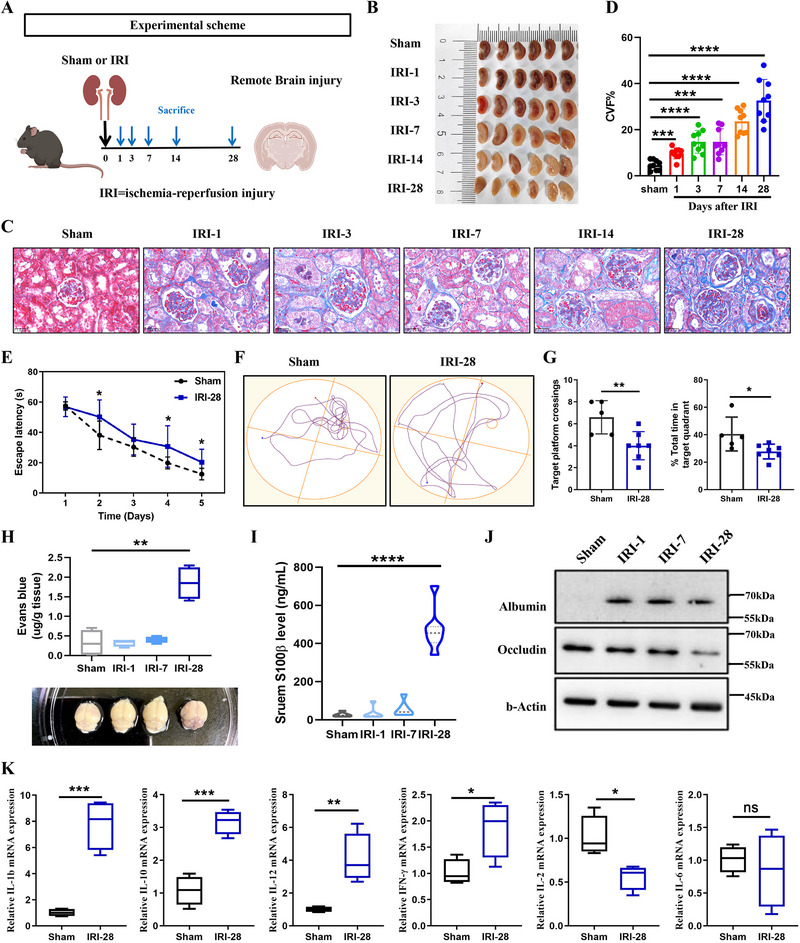
The impact of ischemic kidney injury on cognitive function, BBB permeability, and tissue inflammation. A). Experimental scheme illustrating the timeline of remote cognitive impairment induction and sacrifice at various time points (1, 3, 7, 14, and 28 days) post‐injury. Sham animals were used as controls. B). Representative images of kidney showing the extent of injury at different time points post‐IRI (1, 3, 7, 14, and 28 days), n = 6. C). Masson staining of kidney sections from Sham and IRI groups at 1‐, 3‐, 7‐, 14‐, and 28‐days post‐injury, showing the progression of tissue damage and recovery. D). Quantification of fibrosis scores at different time points post‐IRI, indicating the severity of fibrosis, n = 8. E). Escape latency in the Morris water maze test, comparing Sham and IRI groups at 28 days post‐injury, showing a significant increase in latency in the IRI group, n = 6. F). Representative swim paths in the Morris water maze test for Sham and IRI groups at 28 days post‐injury, highlighting the impaired spatial memory in the IRI group. G). On day 6 of the water maze experiment, the number of times the mice crossed the platform (left) and the time they stayed in the target quadrant (right) was recorded, n = 5 in Sham and n = 7 in IRI‐28 group. H). EB extravasation assay showing increased BBB permeability in the IRI groups compared to the Sham group at 1‐, 7‐ and 28‐days post‐injury, n = 6. I). Quantification of serumS100β levels, a marker of BBB permeability, showing a significant increase in the IRI groups compared to the Sham group, n = 6. J). Western blot analysis of albumin and occludin expression in brain homogenates, with β‐actin as a loading control. The IRI groups show increased albumin levels and decreased occludin levels, indicating BBB disruption, n = 3. K). Quantifying the expression levels of inflammatory factors (IL‐1β, IL‐10, IL‐12, IFN‐γ, IL‐2, IL‐6) in hippocampal tissue showed that compared with the Sham group, the IRI group had an increased inflammatory response in hippocampal tissue, n = 6. Data are represented as mean ± SEM (*p < 0.05, **p < 0.01, ***p < 0.001, ****p < 0.0001).

To explore the transition from AKI to CKD and its impact on renal function and pathology, we developed an AKI mouse model utilizing IRI as depicted in **Figure** **1**A. Our findings revealed a significant decline in renal function at various time points post‐IRI. Visual inspection of kidney tissue photographs revealed substantial ischemic damage during the acute phase, with progressive postoperative damage leading to gradual kidney atrophy and ischemia (Figure 1B). Masson's trichrome staining results indicated that as renal injury progressed, there was a clear observation of renal tubular epithelial cell swelling, degeneration, and necrosis, along with atrophy of renal tubules and loss of brush borders; an increase in renal interstitial collagen fibers and extracellular matrix components was also evident, presenting as multifocal fibrosis (Figure 1C,D). Blood urea nitrogen (BUN) levels significantly increased during the acute phase of kidney injury. Interestingly, at 24 h post‐IRI, we observed a 50% increase in serum BUN levels compared to baseline, which then decreased by 20% as the injury transitioned into the chronic phase over a period of 4 weeks (Figure S1A, Supporting Information). PAS staining results further demonstrated the flattening, shedding, or vacuolization of renal tubular epithelial cells, thickening of the renal tubular basement membrane, and an increase in the extracellular matrix following IRI (Figure S1B, Supporting Information). Moreover, IHC staining for α‐SMA confirmed the enhancement of fibrosis, it increased by ≈30% from the acute to the chronic phase (Figure S1C,D, Supporting Information). Collectively, these findings confirm the successful establishment of an animal model that captures the progression of kidney disease from acute injury to chronic kidney disease.

In the MWM experiment, a widely used test for assessing cognitive function in rodents, we evaluated the cognitive impairment in mice by monitoring their performance in locating a submerged platform.^[^ ^31^ ^]^ Their cognitive abilities were assessed by measuring two key parameters: escape latency, which is the time taken to find the platform, and the swimming path, which indicates the efficiency of their search strategy.^[^ ^32^ ^]^ Our findings revealed that mice subjected to post‐ischemic renal injury demonstrated a markedly longer escape latency, averaging 25s to find the platform, compared to the control group's average of 16s. Additionally, the AKI mice made fewer successful platform crossings, with an average of 3 crossings per trial, whereas the control group achieved an average of 5 crossings per trial. These results indicate that the mice subjected to post‐ischemic renal injury experienced impairments in spatial learning and memory, as evidenced by their increased latency to find the escape platform and reduced ability to navigate the maze effectively (Figure 1E–G).

The BBB is a vital protective mechanism that shields the central nervous system from harmful substances, maintains the stability of the brain microenvironment, and ensures the normal function of nerve cells.^[^ ^33^ ^]^ To assess the permeability of the BBB, we employed Evan's Blue staining, a well‐established method for evaluating BBB integrity and permeability in rodent models.^[^ ^34^ ^]^ Evan's Blue dye, which has a high affinity for plasma albumin, typically cannot cross the BBB under normal conditions. However, when the BBB is compromised, Evan's Blue can penetrate the nervous system, leading to staining. Our brain tissue photographs revealed only faint staining in AKI mouse brain tissue (Figure 1H). To further assess changes in BBB integrity, we homogenized mouse brain tissue and quantified Evan's Blue content using a colorimetric method. The results demonstrated that the Evan's Blue content in the brain tissue of mice subjected to post‐ischemic renal injury was significantly higher than that of the control group, with an increase of 5 times (Figure 1H). similarly, under normal circumstances, the BBB restricts the entry of S100β from brain tissue into the bloodstream. In cases of BBB damage, such as brain injury or inflammation, S100β protein may leak from brain tissue into the bloodstream.^[^ ^35^ ^]^ Therefore, we detected the level of serum S100β in mice using ELISA, and the results showed a significant increase in serum S100β levels in mice subjected to post‐ischemic renal injury (Figure 1I). Western Blot analysis revealed that the albumin levels were markedly elevated and tight‐junction protein Occludin‐5 expression was reduced in post‐AKI mice group compared with controls (Figure 1J). These findings collectively indicate that BBB permeability is increased in post‐AKI mice, indicating a disruption in the protective barrier that could have significant implications for neurological health.

In the hippocampal region, particularly within the CA3 and dentate gyrus (DG) areas, mice in the post‐AKI group exhibited significant hippocampal neuronal damage. This damage was characterized by cellular swelling and deformation, with a reduction in dendritic length or even the complete disappearance of dendritic structures. Furthermore, there was a marked increase in the degree of nuclear condensation, indicative of cellular stress and potential apoptosis (Figure S1E, Supporting Information). To assess the inflammatory response in the brain tissue of mice subjected to post‐ischemic renal injury, we utilized quantitative PCR (Q‐PCR) to measure the expression levels of key inflammatory cytokine genes. Our findings revealed significant upregulation of pro‐inflammatory cytokines in the brain tissue of post‐AKI mice compared to the control group. Specifically, the expression levels of interleukin‐1 β (IL‐1β), IL‐10, IL‐12, and IFN‐γ were notably increased, suggesting heightened inflammatory activity in the brains of post‐AKI mice (Figure 1K). These morphological and molecular alterations likely underlie the spatial learning and memory deficits observed in mice after ischemic renal injury.

### 2.2 Enhanced MDK‐LRP1 Interaction in the Kidney and Hippocampus of Post‐AKI Mice

**Figure 2:**
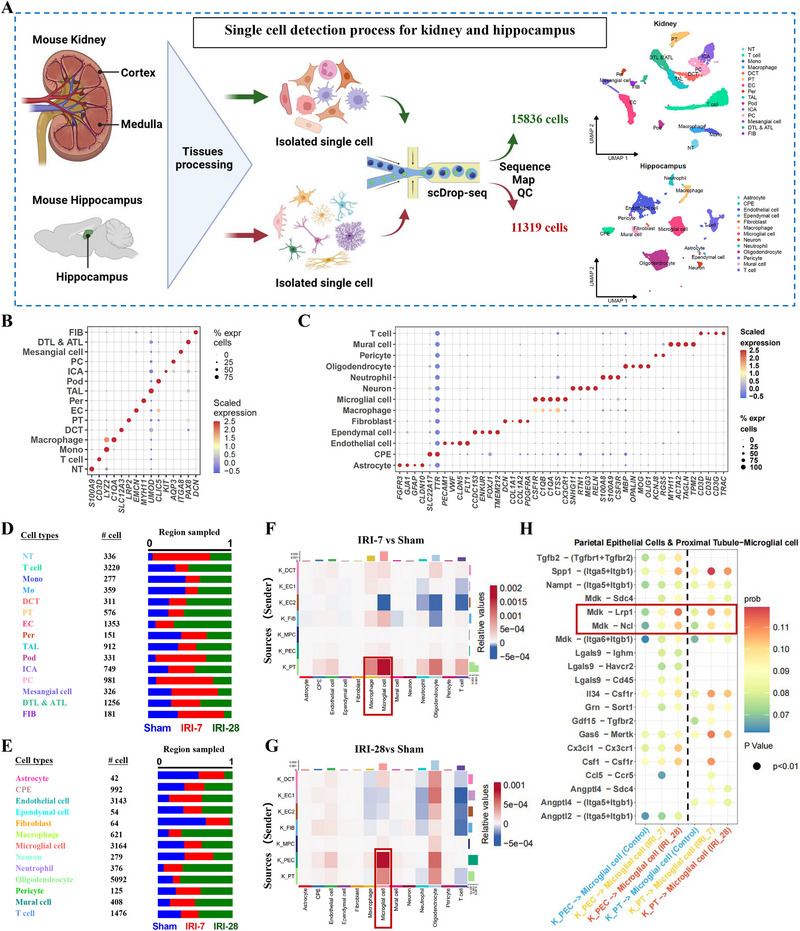
scRNA‐seq maps renal–hippocampal cell shifts and gene networks after ischemic injury. A). Schematic representation of the single‐cell detection process for the kidney and hippocampus. Tissues from the mouse kidney and hippocampus are processed to isolate single cells, which are then subjected to single‐cell RNA sequencing (scDrop‐seq) to analyze gene expression in 15836 cells from the kidney and 11319 cells from the hippocampus. B). A dot plot illustrating the expression levels of key marker genes for various cell types in the kidney. The color gradient represents the expression level of each gene, and the circle size indicates the percentage of each gene expressed across different cells. C). A dot plot depicting the expression levels of marker genes for different cell types in the hippocampus. The color gradient represents the expression level of each gene, and the circle size indicates the percentage of each gene expressed across different cells. D). A bar graph displaying the distribution of cell types in the kidney, with the number of cells for each type indicated. E). A bar graph showing the distribution of cell types in the hippocampus, with the number of cells for each type indicated. F). The heatmaps of interactions between renal cells and hippocampal cells in the IRI‐7 group and the sham surgery group (ligands located in the kidneys and receptors located in hippocampal tissue), with color gradients indicating high or low interaction coefficients, where darker colors denote stronger interactions. G). The heatmaps of interactions between renal cells and hippocampal cells in the IRI‐28 group and the sham surgery group (ligands located in the kidneys and receptors located in hippocampal tissue), with color gradients indicating high or low interaction coefficients, where darker colors denote stronger interactions. H). CellChat presents the ligand‐receptor interaction analysis between renal parietal epithelial cells and renal proximal tubular cells on hippocampal microglial receptors, with color gradients indicating the strength of the interaction. The darker the color, the stronger the interaction.

To delve into the molecular underpinnings of cognitive impairment following kidney injury, we performed scRNA‐seq on kidney and hippocampal tissues from three distinct groups: a control group, mice at an early stage of post‐AKI (7 days post‐IRI, IRI‐7), and mice at a late stage of post‐AKI (28 days post‐IRI, IRI‐28), as depicted in **Figure** **2**A. In our analysis of the molecular profiling of renal and hippocampal cells within the mouse kidneys and hippocampus, a total of 27155 cells passed the quality control filtration and were sequenced. This dataset comprised 15836 cells derived from renal tissue and 11319 cells from hippocampal tissue. The renal cells were categorized into 15 distinct cell clusters, while the hippocampal cells formed 13 separate clusters. We successfully identified all immune and non‐immune cell subtypes present in the hippocampus and renal cells of mice subjected to post‐ischemic renal injury (Figure 2B–E).

Over the past two years, cross‐organ L‐R pairing analysis has emerged as a valuable tool for uncovering the mediators of inter‐organ communication in multi‐organ failure scenarios. To explore potential cell‐cell communication between the kidney and hippocampus, we collected damaged kidney and hippocampus tissues from the above three groups mice for scRNA‐seq. We then applied the machine learning algorithm CellChat for cross‐organ L‐R pairing analysis, focusing on ligands present in the kidney and their corresponding receptors in the hippocampus tissue. The L‐R pairing analysis revealed a significant enhancement in the interaction between renal proximal tubular (PT) cells and hippocampal microglia following kidney injury, compared to the control group (Figure 2F,G; Figure S2, Supporting Information). Further in‐depth analysis indicated that the heparin‐binding growth factor (MDK), secreted by the kidneys, interacts with the low‐density lipoprotein receptor‐related protein 1 (LRP1). The pairing of LRP1 was significantly enhanced, suggesting its potential role in the development of cognitive dysfunction secondary to ischemic renal injury (Figure 2H). This finding provides a potential possibility for further investigation into the molecular mechanisms linking kidney injury to cognitive decline.

### 2.3 Expression Patterns of MDK in Kidney and Hippocampus Tissues

**Figure 3:**
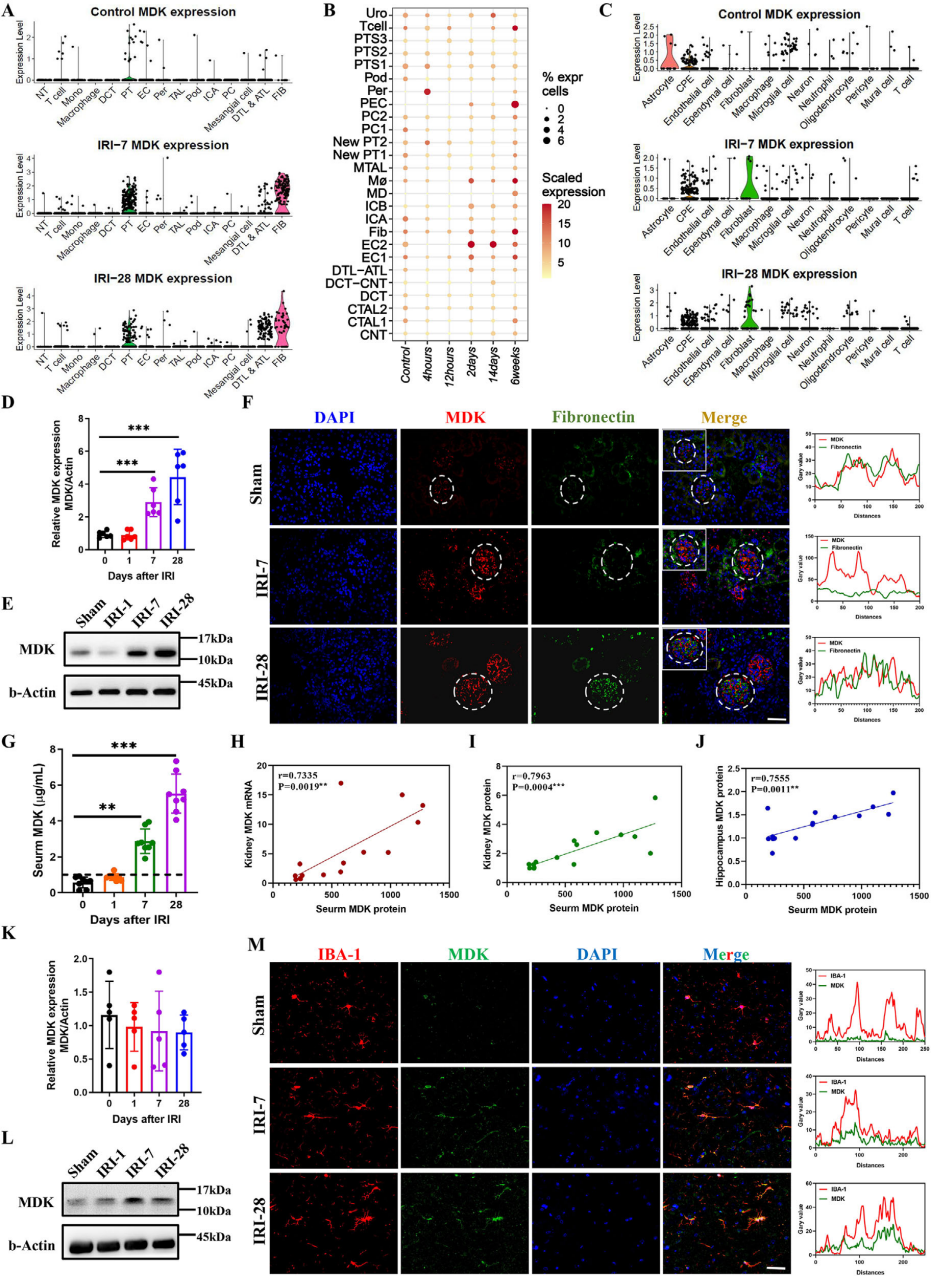
The upregulation of renal MDK expression after ischemic injury may be enriched in brain tissue through circulation. A). Kidney scRNA‐seq: MDK expression after sham or IRI‐7 or IRI‐28. B). Kidney snRNA‐seq: MDK expression after sham or 4 h, 12 h, 2 days, 14 days, and 6 weeks after AKI. C). Hippocampus scRNA‐seq: MDK expression after sham or IRI‐7 or IRI‐28. D). Quantification of relative MDK mRNA expression in kidney at different time points post‐IRI (1, 7, and 28 days) compared to Sham, n = 6. E). Western blot analysis of MDK expression in kidney at 7 and 28 days post‐IRI compared to Sham. β‐Actin is used as a loading control. F). Immunofluorescence staining for MDK (red), fibronectin (green), and DAPI (blue) in Sham, IRI‐7, and IRI‐28 groups. The merged images show the co‐localization of MDK with fibronectin. Scale bar = 50 µm. G). Quantification of relative expression of MDK protein in serum at different time points (1, 7, and 28 days) after IRI compared to sham surgery, n = 8. H). Correlation analysis between serum MDK protein levels and renal MDK mRNA expression levels after IRI. The correlation coefficient (r) and p‐value are displayed. I). Correlation analysis between serum MDK protein levels and renal MDK protein expression levels after IRI. The r and p‐value have been indicated. J). Correlation analysis between serum MDK protein levels and hippocampal MDK protein expression levels after IRI. The r and p‐value have been indicated. K). Quantification of relative MDK mRNA expression in hippocampus at different time points post‐IRI (1, 7, and 28 days) compared to Sham, n = 5. L). Western blot analysis of MDK expression in hippocampus at 7 and 28 days post‐IRI compared to Sham. β‐Actin is used as a loading control. M). Immunofluorescence staining for IBA‐1 (green), MDK (red), and DAPI (blue) in Sham, IRI‐7, and IRI‐28 groups. The merged images show the co‐localization of MDK with IBA‐1. Scale bar = 50 µm. Data are presented as mean ± SEM, with statistical significance indicated by asterisks (**p < 0.01, ***p < 0.001).

To delineate the contribution of MDK to cognitive dysfunction after ischemic renal injury, we profiled MDK mRNA in both kidney and hippocampal tissues throughout the post‐injury time course. Our scRNA‐seq data revealed that MDK mRNA was virtually undetectable in renal cells from the control group. However, on the 7‐day following AKI, MDK expression surged significantly in renal tubular cells and fibroblasts. By the 28th day post‐AKI, MDK was predominantly expressed by the proximal tubules (PT) and the descending thin limb and ascending thin limb (DTL‐ATL) of the nephron (**Figure** **3**A). We conducted a comparative analysis of scRNA‐seq data with previously published single‐nucleus RNA sequencing (snRNA‐seq) data on AKI to validate our findings.^[^ ^36^ ^]^ Consistent with our dataset, the expression levels of MDK mRNA were found to be relatively low in the initial stages. Specifically, four h post‐AKI, there was a significant upregulation of MDK mRNA expression in peritubular cells. This trend continued, and by two days later, MDK mRNA expression was notably elevated in endothelial cells, fibroblasts, and macrophages. After 14 days, endothelial cells maintained high levels of MDK expression, and by six weeks, the expression of MDK mRNA continued to increase across various cell types (Figure 3B). To track the dynamic changes of MDK in our model, we conducted additional experiments inducing renal IRI and assessed MDK gene and protein levels in kidney and hippocampal tissues at 1‐, 7‐, and 28‐days post‐injury. Q‐PCR and Western blot analyses further confirmed that renal MDK expression gradually increased over the 28‐day course after ischemic AKI (Figure 3D,E). IF identification of MDK‐expressing cell types revealed that MDK was primarily expressed by renal tubular cells, aligning with the secretion pattern of Fibronectin, suggesting that MDK is also secreted by fibroblasts (Figure 3F; Figure S3A, Supporting Information).

Furthermore, serum MDK protein levels escalated in tandem with the progression of renal injury (Figure 3G), closely mirroring changes in renal MDK mRNA and protein expression and hippocampal MDK protein levels (Figure 3H–J), suggested that the kidneys are the primary source of hippocampal MDK following renal injury. These findings suggest that MDK secreted after kidney injury may accumulate in hippocampal tissue through the BBB. To verify this possibility, we first analyzed single‐cell sequencing of hippocampal tissue to monitor whether MDK mRNA levels change in parallel with the progression of post‐ischemic renal injury. The results showed that hippocampal tissue showed only low‐level expression of MDK in all cell types, with no significant changes in MDK mRNA expression levels observed on the 7th and 28th days post‐AKI injury, maintaining an overall low expression pattern (Figure 3C,K). However, Western blot results demonstrated a gradual increase in MDK protein expression in hippocampal tissue on the 1st, 7th, and 28th days following kidney injury (Figure 3L). IF staining results indicated that MDK protein predominantly co‐localized with IBA‐1, a microglia marker, in hippocampal tissue (Figure 3M), hinting at a potential role in mediating functional changes in microglia. Collectively, these findings suggest that kidney‐derived MDK contributed to cognitive dysfunction after ischemic renal injury.

### 2.4 Activation of Hippocampal Microglia in Mice Subjected to Post‐Ischemic Renal Injury

**Figure 4:**
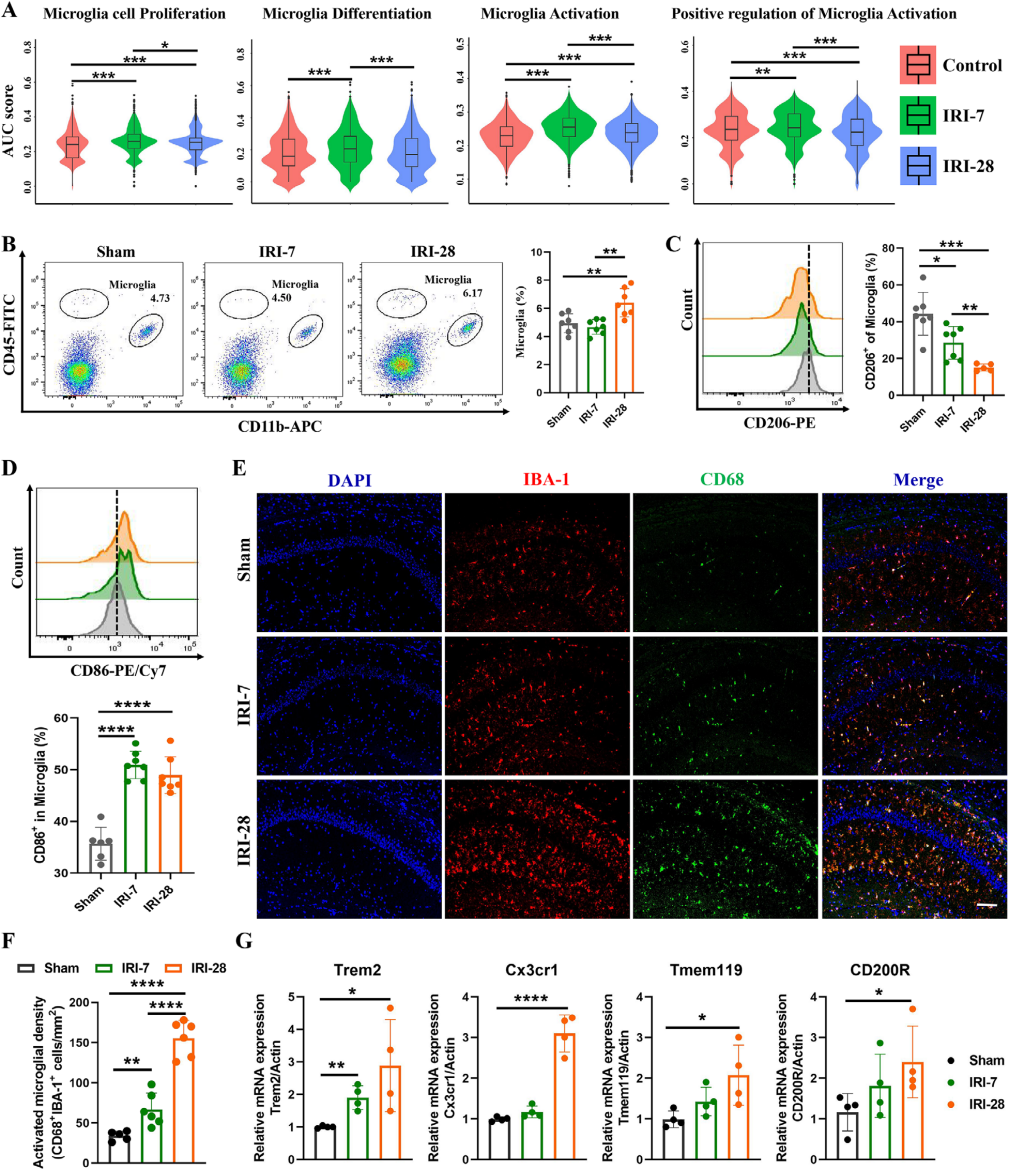
Activation of microglia after ischemic chronic kidney injury, exhibiting a chronic inflammatory phenotype. A). Violin plots showing the AUC score of microglia cell proliferation, microglia differentiation, microglia activation, and positive regulation of microglia activation in control, IRI‐7, and IRI‐28 groups. B). Flow cytometry analysis of microglia in control, IRI‐7, and IRI‐28 groups, n = 7. C). Flow cytometry analysis of CD206‐positive microglia in control, IRI‐7, and IRI‐28 groups. The histogram shows the percentage of CD206‐positive cells within the microglia population, n = 7. D). Flow cytometry histogram showing the percentage of CD86‐positive cells within the microglia population in Sham, IRI‐7, and IRI‐28 groups, n = 7. E). Immunofluorescence staining for DAPI (blue), IBA‐1 (red), and CD86 (green) in Sham, IRI‐7, and IRI‐28 groups. The merged images show the co‐localization of CD86 with IBA‐1‐positive microglia. Scale bar = 50 µm. F). Bar graphs quantifying the number of activated microglia (IBA‐1^+^) and the density of CD86^+^ cells in the IRI groups at 7‐ and 28‐days post‐injury compared to Sham, n = 6. G). Bar graphs showing the relative mRNA expression levels of Trem2, Cx3cr1, Tmem119, and Cd200r in control, IRI‐7, and IRI‐28 groups, n = 4. Data are presented as mean ± SEM, with statistical significance indicated by asterisks (*p < 0.05, **p < 0.01, ***p < 0.001, ****p < 0.0001).

The co‐localization of MDK and IBA‐1 observed earlier suggests a potential impact on the phenotype and activation of microglia. Consequently, we scrutinized the functional alterations in microglia through single‐cell sequencing and discovered that the proliferation, differentiation, and activation of microglia in mice subjected to post‐ischemic renal injury were markedly elevated compared to the control group (**Figure** **4**A). The scRNA‐seq identified various genes associated with microglial activation and function across different cell clusters, such as TMEM119, SIGLECH, CTSS, CX3CR1, AIF1, and TYROBP (Figure S4A, Supporting Information). These genes serve as markers for microglia and reflect their activation status, pointing to functionally diverse microglial subpopulations. Further analysis via Gene Ontology (GO) revealed that, compared to the control group, the IRI‐7 group exhibited significant enrichment of biological processes (BPs) related to cell migration and microglial responses to external stimuli (Figure S4B, Supporting Information). In contrast, the IRI‐28 group showed marked enrichment in processes regulating gliogenesis, immune responses, and leukocyte migration (Figure S4C, Supporting Information). These results suggest that microglia in mice subjected to post‐ischemic renal injury undergo activation and polarization.

Additionally, we employed flow cytometry to assess the number and phenotypic changes of hippocampal microglia in the Sham, IRI‐7, and IRI‐28 mouse groups. We observed a notable increase in the microglial population within the hippocampus of post‐AKI mice, indicating an immune response in this brain region (Figure 4B). A detailed analysis of microglial phenotypes revealed a predominance of the M1 phenotype, associated with pro‐inflammatory actions, in the hippocampus of post‐AKI mice. Conversely, the M2 phenotype, linked to anti‐inflammatory and tissue repair processes, showed a progressive decline (Figure 4C,D). This phenotypic shift suggested that microglia remain in a sustained pro‐inflammatory state after ischemic renal injury.

Immunofluorescence staining corroborated these findings, showing not only an increase in the number of microglia but also elevated CD86 expression in the hippocampus of mice subjected to post‐ischemic renal injury. CD86 is a marker of microglial activation, and its increased expression further confirms the pro‐inflammatory state of these cells. Notably, the proportion of CD86‐positive microglia in the IRI‐28 group was more than three times higher than in control, providing clear evidence of progressive microglial activation after ischemic renal injury (Figure 4E,F).

Moreover, we investigated the expression levels of genes related to microglial activation in hippocampal tissue. The results showed that the expression of Trem2, Cxc3r1, Tmem19, and CD200R was significantly upregulated in post‐AKI mice compared to the control group (Figure 4G). These genes are involved in microglial activation and function, and their increased expression supports the notion that microglia in the hippocampus of mice subjected to post‐ischemic renal injury are in an activated, pro‐inflammatory state. The sustained pro‐inflammatory state of microglia could contribute to synaptic loss and impaired neural function, which are key factors in the development of cognitive impairments in post‐AKI patients. Further research into the specific mechanisms of microglial activation and the potential therapeutic targets to mitigate this response is warranted.

### 2.5 MDK is Internalized into Microglia through LRP1 Mediation

MDK, a well‐characterized heparin‐binding cytokine, is secreted into the extracellular environment following the cleavage of its signal peptide. Extensive research has demonstrated that MDK can be internalized into cells that do not endogenously express MDK via receptor‐mediated endocytosis pathways. To explore the potential link between hippocampal microglial activation and local MDK protein accumulation, we cultured in vitro cell lines of HK2, NRK‐52e, and NRK‐49F that were genetically modified to overexpress MDK (Figure S5A,B, Supporting Information).

**Figure 5:**
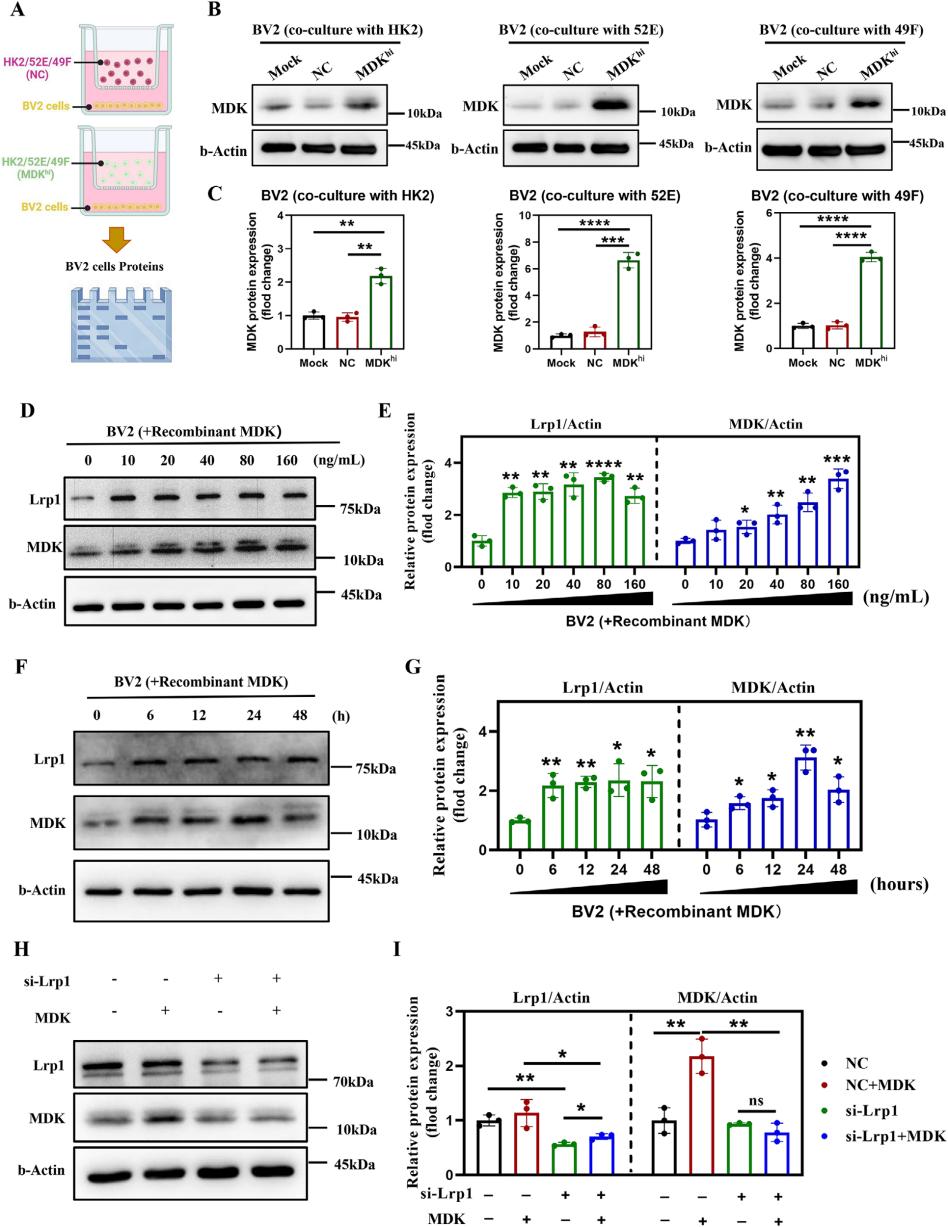
MDK is internalized into microglia by binding to Lrp1. A). Schematic diagram of co culture system between BV2 cells and HK2/NRK‐52E/NRK‐49F cells, where HK2/NRK‐52E/NRK‐49F cells were pre transfected with midkine plasmid to induce high expression of MDK (MDK^hi^). B). Western blot analysis showed that compared to the untreated group and NC group, the expression of MDK in BV2 cells significantly increased after co culturing with HK2 cells, 52E cells, or 49F cells of MDK^hi^ for 48 h. β‐actin is used for load control. C). Bar graphs quantifying MDK protein expression in BV2 cells after co‐culture with HK2, 52E, or 49F cells, n = 3. D). Western blot analysis of Lrp1 and MDK expression in BV2 cells treated with increasing concentrations of rMDK (0, 10, 20, 40, 80, 160 ng mL^−1^) for 48 h. β‐Actin is used as a loading control. E). Bar graphs showing the relative protein expression of Lrp1 and MDK in BV2 cells treated with different concentrations of rMDK, n = 3. F). Western blot analysis of Lrp1 and MDK expression in BV2 cells treated with 100 ng mL^−1^ rMDK over time (0, 6, 12, 24, 48 h). β‐Actin is used as a loading control. G). Bar graphs quantifying the relative protein expression of Lrp1 and MDK in BV2 cells treated with rMDK at different time points, n = 3. H). Western blot analysis of Lrp1 and MDK expression in BV2 cells transfected with si‐Lrp1 and treated with rMDK. β‐Actin is used as a loading control. I). Bar graphs showing the relative protein expression of Lrp1 and MDK in BV2 cells transfected with si‐Lrp1 or si‐NC (negative control) and treated with rMDK, n = 3. Data are presented as mean ± SEM, with statistical significance indicated by asterisks (*p < 0.05, **p < 0.01, ***p < 0.001, ****p < 0.0001).

We then collected the supernatants from these cell cultures, which contained secreted MDK, to use for culturing microglial cell lines (**Figure** **5**A). Our findings revealed that when microglia were treated with supernatants from MDK‐overexpressing cells, there was a substantial increase in intracellular MDK levels, with at least a 3‐fold increase observed at 24 h post‐treatment (Figure 5B,C). To further investigate the dynamics of MDK internalization, we generated a recombinant MDK (rMDK) protein in vitro and introduced it into the culture medium of microglial cells. The results demonstrated a time‐dependent and dose‐dependent increase in intracellular MDK levels. Specifically, the addition of recombinant MDK to microglial cultures induced a dose‐dependent response, with a 2.2‐fold increase at a concentration of 20 ng/mL and a 3.0‐fold increase at 80 ng/mL after 24 h of treatment. Moreover, the level of MDK in microglia increased by approximately 2.0‐fold within just 6 h of treatment with MDK (Figure 5D–G). Interestingly, we also observed that the protein expression level of LRP1 on microglia increased when cells were exposed to a short period (6 h) and a low concentration (10 ng mL^−1^) of recombinant MDK protein. This suggests that the upregulation of LRP1 may serve as an adaptive response to enhance the transport efficiency of MDK (Figure 5D–G).

To determine if the internalization of MDK is mediated by its receptor LRP1, we genetically deleted LRP1 in microglia using CRISPR/Cas9 technology and subsequently treated these cells with recombinant MDK protein (Figure S5C,D, Supporting Information). The results demonstrated that the absence of LRP1 almost completely blocked the endocytosis of MDK, underscoring the pivotal role of LRP1 in MDK uptake (Figure 5H,I). Collectively, these findings indicate that MDK can be efficiently internalized into microglia via LRP1‐mediated endocytosis, potentially leading to morphological and functional changes in these cells. This mechanism may underlie the neuroinflammatory observed in post‐AKI cognitive impairment.

In addition to LRP1, MDK has multiple receptors, including heparan sulfate syndecan‐3 (SDC3), integrins (such as ItgA4, ItgA6, Itgb1, Itgb2 and Itgb3), Notch receptor 2 (Notch2), anaplastic lymphoma kinase (ALK), and protein tyrosine phosphatase receptor type Z1 (PTPRZ1).^[^ ^37^ ^]^ They binding to MDK can participate in various biological processes. We further analyzed single‐cell data from the hippocampal tissue of AKI mice and found that, compared with ItgA6, Itgb1, and Itgb2, the expression levels of other MDK receptors in microglia were extremely low or nearly undetectable. Among these three receptors, only the expression levels of ItgA6 and Itgb1 gradually increased with the progression of post‐AKI, whereas the expression of Itgb2 progressively decreased with AKI development (Figure S6A,B, Supporting Information). To further elucidate whether ItgA6 and Itgb1 are involved in mediating the internalization of MDK, we designed siRNAs targeting these receptors and confirmed their ability to inhibit the expression of ItgA6 and Itgb1 in microglia (Figure S6C,D, Supporting Information). Subsequently, we treated these cells with recombinant MDK protein after inhibiting ItgA6 and Itgb1. The results showed that the absence of ItgA6 and Itgb1 did not affect the endocytosis of MDK (Figure S6E,F, Supporting Information) and did not alter MDK‐mediated microglial activation and inflammatory responses (Figure S6G,H, Supporting Information), highlighting the critical role of LRP1 in MDK uptake.

### 2.6 MDK‐LRP1 Upregulated P2ry12 Expression Mediates the Activation of Microglia Cells

**Figure 6:**
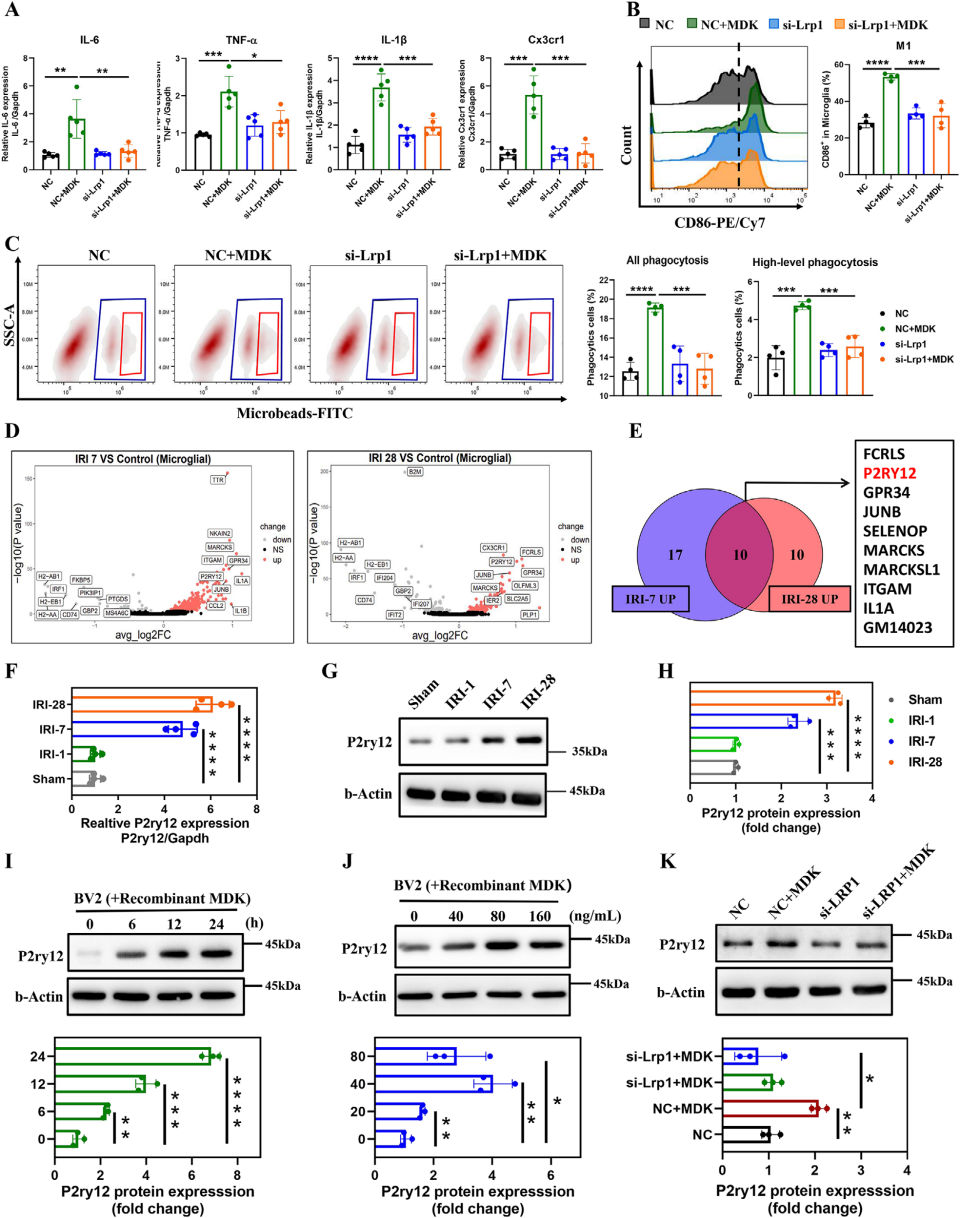
MDK‐Lrp1 upregulates the expression of P2ry12 in microglia and activate their phagocytic activity and inflammatory cytokine expression. A). Bar graphs showing the relative mRNA expression levels of inflammatory cytokines in BV2 cells transfected with si‐Lrp1 or si‐NC (negative control) and treated with rMDK, n = 5. B). Flow cytometry histograms and bar graphs displaying the percentage of all phagocytosis and high‐level phagocytosis in BV2 cells transfected with si‐Lrp1 or si‐NC and treated with rMDK, n = 4. C). Beads‐FITC uptake assay in BV2 cells transfected with si‐Lrp1 or si‐NC and treated with rMDK, n = 4. D). The volcano plot displays genes with differential changes in hippocampal microglia between IRI‐7 and IRI‐28 groups compared to the Sham group, with red: upregulation, gray: downregulation, and black: no change. E). Venn diagram comparing the upregulated genes in IRI‐7 and IRI‐28 groups, with a focus on genes related to activation of microglia. F). Bar graph showing the relative P2ry12 mRNA expression in hippocampus at IRI‐7 and IRI‐28 groups compared to Sham, n = 4. G). Western blot analysis of P2ry12 expression in hippocampus at 1, 7 and 28 days post‐IRI compared to Sham. β‐Actin is used as a loading control. H). Bar graph showing the relative P2ry12 protein expression in hippocampus at IRI‐7 and IRI‐28 groups compared to Sham,n = 3. I). Western blot analysis of P2ry12 protein expression in BV2 cells treated with rMDK over time. Bar graphs quantifying the relative protein expression of P2ry12 in BV2 cells treated with rMDK at different time points, n = 3. J). Western blot analysis of P2ry12 expression in BV2 cells treated with increasing concentrations of rMDK (0, 10, 20, 40, 80, 160 ng mL^−1^) for 48 h. β‐Actin is used as a loading control. Bar graphs showing the relative protein expression of P2ry12 in BV2 cells treated with different concentrations of rMDK, n = 3. K). Western blot analysis of P2ry12 expression in BV2 cells transfected with si‐Lrp1 and treated with rMDK. β‐Actin is used as a loading control. Bar graphs showing the relative protein expression of P2ry12 in BV2 cells transfected with si‐Lrp1 or si‐NC and treated with rMDK, n = 3.Data are presented as mean ± SEM, with statistical significance indicated by asterisks (*p < 0.05, **p < 0.01, ***p < 0.001, ****p < 0.0001).

To delve deeper into the effects of MDK internalization on microglial activation, we examined the expression of inflammatory factors in microglia following MDK treatment. Our results demonstrated significant upregulation of key pro‐inflammatory genes, including IL‐6, TNF‐α, IL‐1β, and Cx3cr1, in microglia post‐MDK treatment, indicating a robust inflammatory response. However, when LRP1 expression was inhibited, these gene expression levels remained unresponsive to MDK stimulation, suggesting that LRP1 is critical for MDK‐induced inflammatory signaling (**Figure** **6**A). Flow cytometry analysis revealed that MDK treatment promoted microglial polarization toward the M1 phenotype, a pro‐inflammatory state, and this effect was abrogated by inhibiting LRP1 expression (Figure 6B). Furthermore, in vitro experiments using fluorescent microbeads showed that MDK treatment significantly enhanced the microglial ability to engulf particles. Conversely, inhibiting LRP1 expression not only blocked MDK internalization but also suppressed the activation of microglial phagocytosis (Figure 6C). These findings collectively suggest that MDK‐LRP1 signaling plays a pivotal role in mediating the pro‐inflammatory activation and heightened phagocytic function of microglia.

To elucidate the molecular mechanisms underlying MDK‐LRP1 signaling in microglial activation, we analyzed upregulated genes in hippocampal microglia from mice in the IRI‐7 and IRI‐28 groups compared to the Sham group using single‐cell sequencing data. We observed a marked increase in P2ry12 mRNA expression in hippocampal microglia during both the early and late stages of AKI (Figure 6D). This upregulation was confirmed by Q‐PCR and Western blot analysis (Figure 6F–H). P2ry12 is a G‐protein‐coupled receptor that plays a crucial role in microglial activation, facilitating microglial migration, chemotaxis, and the regulation of neuroinflammation during inflammatory responses. We found that P2ry12 expression increased in a time‐ and dose‐dependent manner following MDK treatment (Figure 6I–K). Additionally, interfering with LRP1 expression significantly attenuated the MDK‐induced activation of P2ry12 (Figure 6I–K). These results indicate that MDK‐LRP1 signaling may mediate the pro‐inflammatory activation and enhanced phagocytic function of microglia by upregulating P2ry12. This mechanism provides valuable insights into how MDK drives neuroinflammation and cognitive dysfunction after AKI.

### 2.7 Inhibition of Renal MDK Alleviates AKI‐Induced Cognitive Impairment and Activation of Microglia

**Figure 7:**
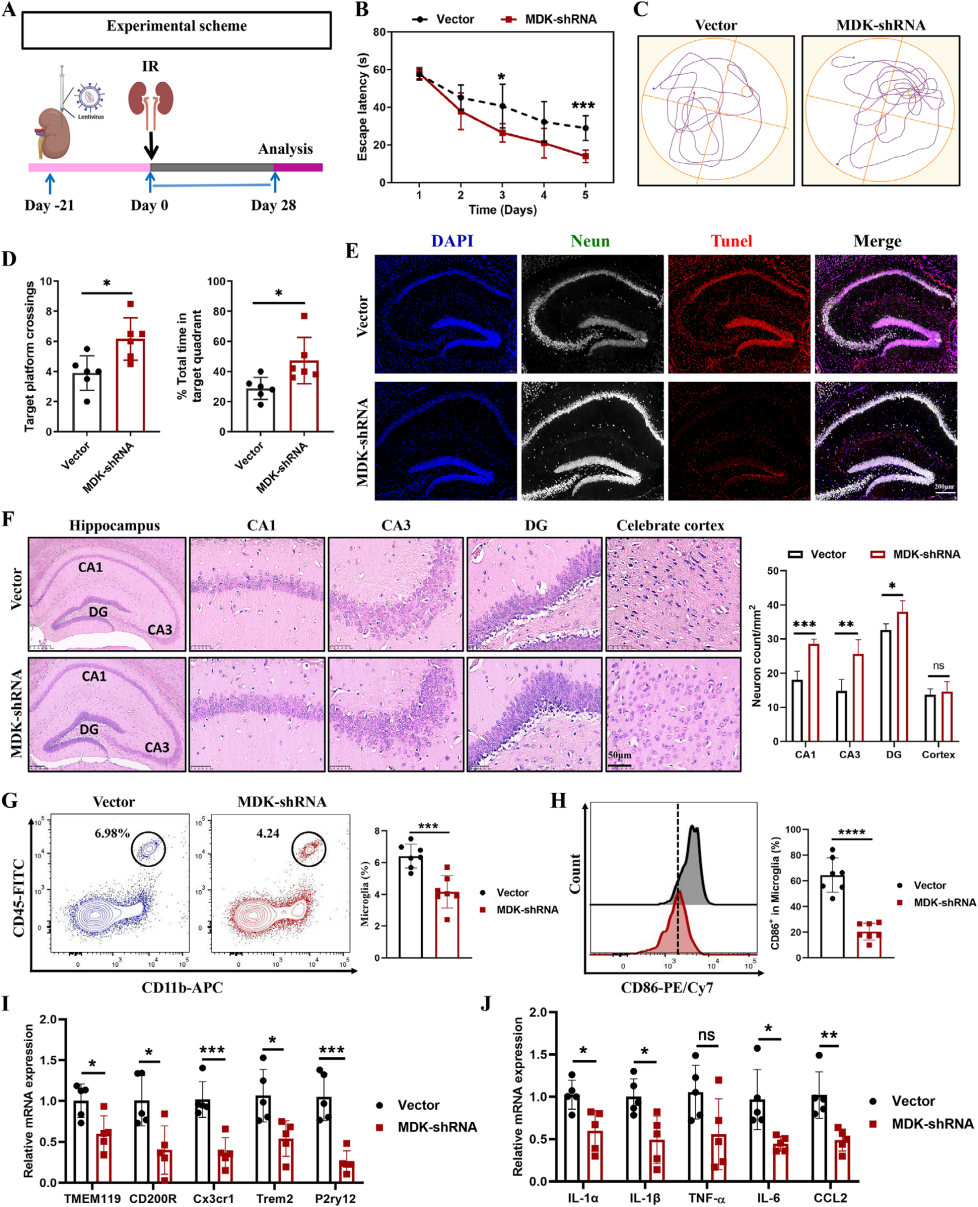
Inhibition of renal MDK expression alleviates cognitive impairment and activation of microglia following AKI. A). Experimental plan: On the first day, AAV virus (MDK‐shRNA) carrying targeted MDK shRNA and vector virus (Vector) were injected locally into the kidneys to knock down the expression of MDK. After 21 days of stable virus infection, an IRI model was constructed and tested 28 days after kidney injury. B). Escape latency in the MWM test, comparing MDK‐shRNA and vector group, showing a significant decrease in latency in the MDK‐shRNA group mice, n = 6. C). Representative swim paths in the Morris water maze test for MDK‐shRNA and vector group, highlighting the improved spatial memory in the MDK‐shRNA group mice. D). On day 6 of the water maze experiment, the number of times the mice crossed the platform (left) and the time they stayed in the target quadrant (right) was recorded, n = 6. E). The Tunnel staining of mouse hippocampal tissue slices in the figure shows the level of neuronal apoptosis, while immunofluorescence staining of DAPI (blue), NeuN (green), and Tunnel (red). The merged image displays the common localization of these markers. Scale bar = 50 µ m. F). H&E staining of brain tissue sections was performed to showing the extent of tissue damage, including CA1, CA3, DG and cortex. Scale bars represent 250 µm for hippocampal tissue and 100 µm for CA3 and Cortex regions, n = 6. G). Flow cytometry analysis of microglia (CD45^hi^CD11b^+^) in the hippocampus at Vector and MDK‐shRNA group mice, with the percentage of microglia, n = 7. H). Flow cytometry histogram showing the CD86 expression on microglia in the hippocampus of Vector and MDK‐shRNA group mice, n = 7. I). Bar graphs showing the relative mRNA expression levels of inflammatory markers (TMEM119, CD200R, Cx3cr1, Trem2, P2ry12) in the hippocampus of Vector and MDK‐shRNA group mice, n = 5. J). Bar graphs showing the relative mRNA expression levels of cytokines (IL‐1α, IL‐1β, TNF‐α, IL‐6, CCL2) in the hippocampus of Vector and MDK‐shRNA group mice, n = 5. Data are presented as mean ± SEM, with statistical significance indicated by asterisks (ns = not significant, *p < 0.05, **p < 0.01, ***p < 0.001, ****p < 0.0001).

To determine how renal MDK expression contributes to cognitive impairment following AKI, we locally injected adenoviruses carrying MDK‐shRNA (MDK‐shRNA) and a control vector virus (Vector) into the kidneys of mice, followed by the induction of IRI (**Figure** **7**A). We first confirmed that the injection of these viruses effectively inhibited the expression of MDK at both the gene and protein levels in renal tissue (Figure S7A,B, Supporting Information). Concurrently, there was a significant reduction in serum MDK levels and the accumulation of MDK protein in hippocampal tissue (Figure S7C, Supporting Information).

In the MWM experiment, mice with post‐ischemic renal injury injected with the control viral vectors had an escape latency of ≈26 s, whereas the inhibition of renal MDK expression reduced the time to climb onto the platform to ≈15 s (Figure 7B). Furthermore, the number of successful platform crossings and the duration of stay in the target quadrant were significantly increased in the MDK‐shRNA group compared to the vector group. The number of platform crossings in the MDK‐shRNA group increased by 40% compared to the vector group, and the time spent in the target quadrant was 1.5 times (Figures 7C,D). These findings suggest that the inhibition of renal MDK expression ameliorates spatial learning and memory impairments in AKI mice. Although we cannot formally rule out subtle renal benefits that contribute to cognitive improvement, serum urea and creatinine were already within normal ranges during behavioral testing (IRI‐28), indicating that the observed cognitive protection is unlikely to be solely a result of further improvement in kidney function.

Additional testing revealed that inhibiting renal MDK expression significantly reduced hippocampal cell apoptosis (Figure 7E; Figure S7D, Supporting Information), and mitigated the loss of neurons in the CA1, CA3, and DG regions (Figure 7F). Flow cytometry analysis demonstrated that renal MDK knockdown reversed the AKI‐induced increase in hippocampal microglial numbers, yielding a 30% reduction compared with control virus‐treated mice (Figure 7G). Moreover, the M1 polarization of microglia decreased nearly 60% (Figure 7H). moreover, the expression of activation marker genes (Tmem119, CD200R, Cx3cr1, Tmem2, P2ry12) and inflammatory factors of microglia (IL‐1a, IL‐1b, TNF‐a, IL‐6, CCL2) in hippocampal tissue was alleviated (Figure 7I,J). Collectively, these results indicate that inhibiting the expression of renal MDK alleviates cognitive dysfunction and reduces the activation of microglia secondary to AKI.

## 3 Discussion

The current study offers a comprehensive exploration of the role of MDK in mediating cognitive impairment and microglial activation in AKI. Our findings reveal a significant upregulation of MDK in renal cells following IRI, with a concurrent increase in MDK levels in the peripheral blood and hippocampus of mice with post‐ischemic renal injury. This elevation in MDK correlates with an increase in activated microglia in the hippocampus and an upregulation of P2ry12 expression, suggesting a potential link between MDK, microglial activation, and cognitive deficits after ischemic renal injury. Furthermore, our study demonstrates that inhibiting renal MDK expression alleviates cognitive impairment and reduces microglial activation, highlighting the therapeutic potential of targeting the MDK‐LRP1 pathway in AKI‐related cognitive decline.

MDK is a highly expressed neurotrophic growth factor during embryonic development, with important functions related to growth, proliferation, survival, migration, angiogenesis, reproduction, and repair.^[^ ^38^, ^39^ ^]^ Previous studies have shown that MDK is associated with various pathological conditions, including kidney injury and central nervous system diseases.^[^ ^40^ ^]^ The latest research demonstrated that MDK mainly expressed by CD31^+^ACTA2^+^ ECs going through partial EndMT contributes greatly to myofibroblasts by spatial and single cell transcriptomics MDK promotes renal fibrosis by stabilizing C/EBP β and promoting endothelial mesenchymal transition.^[^ ^17^ ^]^ In this study, we also confirmed that the expression of MDK in renal cells increased after IRI, indicating a close correlation between MDK and the progression of renal injury. Furthermore, our observation of elevated serum MDK levels in mice subjected to post‐ischemic renal injury is corroborated by Şalaru et al., who reported increased MDK levels in advanced AKI patients, indicating its potential as a biomarker for renal injury.^[^ ^41^ ^]^ This underscores the relevance of MDK in AKI to CKD pathophysiology and its association with disease severity.

In our study, we observed an elevation in MDK levels in the serum and hippocampus of mice subjected to post‐ischemic renal injury, suggesting that MDK could serve not only as a biomarker for kidney injury but also may be implicated in cognitive impairment. In the context of cognitive impairment, Takada et al. suggested an association between MDK and cognitive deficits by showing that disruption of the MDK gene reduced traumatic brain injury through the modulation of neuroinflammation.^[^ ^19^ ^]^ Our study extends these findings by elucidating a direct link between MDK upregulation, microglial activation, and cognitive deficits in AKI. This connection is further supported by the understanding that MDK, as a heparin‐binding growth factor, plays a role in neuro‐immune crosstalk under pathological conditions, as indicated by its increased levels in the periphery and central nervous system (CNS) during various pathological states.^[^ ^42^ ^]^ This suggests that MDK is not only a mediator of disease processes within specific compartments but also a facilitator of neuro‐immune cell‐to‐cell communication, which is crucial in the context of AKI‐related cognitive impairment.

A recent study by Abdulmalek et al. (2024) showed that a brief, learning‐evoked hippocampal surge of MDK enhances memory, whereas our IRI model reveals the opposite phenotype.^[^ ^43^ ^]^ Reconciling these findings, we propose that the cognitive outcome is dictated by the intrinsic properties of MDK itself. A brief, learning‐evoked hippocampal pulse of MDK (<24 h) enhances memory, yet prolonged systemic exposure in maladaptive repair after acute kidney injury does the opposite. In the maladaptive repair after acute kidney injury setting, renal‐derived MDK remains elevated for weeks, bathing the forebrain at concentrations >100 ng mL^−1^‐far above the 10–20 ng mL^−1^ required for synaptic potentiation.^[^ ^44^ ^]^ This excess ligand shifts receptor preference from TrkB to RPTPζ, triggering PTEN‐mediated Akt suppression and synaptic loss.^[^ ^45^ ^]^ Continuous spill‐over of MDK also engages microglial RPTPζ, establishing a self‐sustained inflammatory loop that down‐regulates PSD‐95 and synaptophysin.^[^ ^46^ ^]^ Thus, the same molecule that fosters plasticity when neuron‐restricted and transient becomes neurotoxic when chronically abundant and systemically delivered.

In our study, cross organ L‐R analysis based on single‐cell sequencing and CellChat was used, and the results showed an enhanced interaction between kidney cells and hippocampal microglia after kidney injury. This approach has been previously validated in studies such as those by Colonna^[^ ^23^ ^]^ and Butovsky,^[^ ^47^ ^]^ who emphasized the responsiveness of microglia to peripheral signals in neurodegeneration. This demonstrates the practicality of cross organ ligand receptor pairing analysis in deciphering multiple organ failure scenarios. Based on this understanding, we have demonstrated a possible sharing mechanism involving the regulation of microglial function by peripheral factors, which may be key to neuroinflammation and cognitive impairment associated with chronic kidney disease. In this study, we elucidated that MDK‐LRP1 interaction is significantly enhanced after AKI. This finding is particularly significant as it reveals a potential pathway where peripheral immune signals, such as MDK, can penetrate the compromised BBB, triggering responses from resident cells in the CNS and perpetuating inflammatory processes. This provides new insights into the cross‐organ communication between the kidney and the hippocampus after AKI. It has been reported that hippocampal astrocytes can synthesize and release MDK, thereby directly modulating synaptic plasticity and cognitive performance.^[^ ^48^ ^]^ Although the L‐R pairing approach reduces systemic MDK confounding, it does not exclude the possibility that MDK synthesized locally by hippocampal astrocytes contributes to cognitive performance. While our kidney‐restricted knockdown experiment indicates that most hippocampal MDK is kidney‐derived in mice subjected to post‐ischemic renal injury, future studies using astrocyte‐specific Mdk knockout or intrahippocampal neutralization will be required to quantify the relative contributions of central versus peripheral MDK.

It has been demonstrating that the interaction between MDK and its receptor Lrp1 is significantly positively correlated with the infiltration of M2 macrophages in the tumor microenvironment, while negatively correlated with the infiltration of M1 macrophages. The findings collectively suggest that MDK‐LRP1 expression promotes M2 macrophage polarization, contributing to the establishment of an immunosuppressive microenvironment.^[^ ^49^ ^]^ Previous studies have indicated that LRP1 can modulate the immune response of microglia by regulating the JNK and NF‐κB signaling pathways.^[^ ^50^ ^]^ The downregulation of LRP1 leads to the activation of NF‐κB in the absence of inflammatory stimuli and enhances LPS‐induced NF‐κB activity, thereby influencing the activation status of microglia. Collectively, these findings suggest that the MDK‐LRP1 axis operates through distinct mechanisms in different pathological contexts, providing into its role in cross‐organ communication and AKI‐associated neuropathy.

P2ry12, a G protein‐coupled purinergic receptor, plays a pivotal role in a spectrum of physiological and pathological processes, including platelet aggregation, immune response, neuroprotection, inflammation, and cell proliferation.^[^ ^51^, ^52^, ^53^ ^]^ Its significance in the central nervous system is underscored by its involvement in neurotransmission and its links to Alzheimer's disease, atherosclerosis, and other conditions.^[^ ^54^, ^55^ ^]^ P2ry12's participation in excitatory synaptic transmission within the spinal cord's layer II neurons during neuropathic pain in rodents underscores its role in microglial activation.^[^ ^56^, ^57^ ^]^ When neurons sustain damage, the activation of the P2ry12 in microglia by outward potassium currents is necessary, highlighting a direct connection between P2Y12 activation and microglial function.^[^ ^58^ ^]^ In the context of cognitive impairment associated with AKI, our study revealed that MDK treatment upregulated P2ry12 expression in microglia. MDK is internalized into microglia via its receptor Lrp1, then activates P2ry12 expression, promoting the pro‐inflammatory activation and phagocytic function of microglia. This suggests that MDK is a crucial mediator of microglial pro‐inflammatory activation and enhanced phagocytic function, offering insights into the neurotoxic effects observed in various pathologies, including AKI‐related cognitive decline.

In conclusion, our study contributes the MDK‐LRP1 axis as a mechanistic bridge between renal injury, hippocampal neuroinflammation and cognitive decline after AKI, offering new therapeutic targets for post‐AKI cognitive impairment. The consistency of our findings with previous research, along with the identified role of MDK in mediating neuro‐immune interactions, positions MDK as a key molecule in the pathogenesis of AKI‐related complications and a potential therapeutic target. Future research should focus on validating these findings in clinical settings and exploring the therapeutic potential of targeting the MDK‐LRP1 pathway in AKI management.

## 4 Experimental Section

### Animal Experiment

Eight‐week‐old SPF‐grade C57BL/6 mice were purchased from the Model Animal Institute of Nanjing University. The experiments on mice were approved by Institutional Animal Care and Use Committee, Nanjing University, and all experiments were performed in accordance with relevant guidelines and regulations. Mice were acclimatized in a specific pathogen‐free (SPF) facility for one week under a 12‐hour reverse light/dark cycle.

For kidney injury Model: Mice were randomly divided into six groups (n = 10 per group): Sham, IRI‐1, IRI‐3, IRI‐7, IRI‐14, IRI‐28 model. Acute kidney injury was induced using a unilateral renal ischemia‐reperfusion injury (IRI) model. Mice were anesthetized with 2% isoflurane, and anesthesia depth was monitored by observing respiratory rate and corneal reflex. The left renal vessels were clamped for 45 min to induce ischemia, confirmed by the color change of the kidney from red to purple‐black. Sham mice underwent a similar procedure without clamping. Body temperature was maintained between 36.5–37.5 °C using a heating pad.

For renal MDK Knockdown Model: A shRNA adenovirus targeting mouse kidney MDK was constructed and administered via three local injections (2 µL each) into the renal cortex at a titer of 1×10.^[^ ^10^ ^]^ The injections were evenly spaced to ensure uniform distribution. The knockdown efficiency was verified using qRT‐PCR, western blot, and immunofluorescence assays at 48 h post‐injection. Subsequent experiments were conducted among 21 days post‐injection to assess hippocampus‐dependent learning and cognitive functions, neuronal injury, and microglial activation.

### Hematoxylin and Eosin (H&E) Staining

Kidney and brain were harvested from euthanized mice and fixed in 4% paraformaldehyde (PFA) for at least 12 h at 4 °C. Fixed kidney or brain were dehydrated through a graded series of ethanol, cleared in xylene, and embedded in paraffin. Sections (3 µm thick) were cut using a microtome and mounted on glass slides. Images were captured using a microscope at a standardized magnification. The extent of renal injury was quantified using a semi‐quantitative scoring system.

### Masson's Trichrome Staining and fibrosis quantification

Begin by deparaffinizing the tissue sections and rehydrating them through a graded series of ethanol and then wash in distilled water. For tissues fixed with formalin, re‐fixed the sections in Bouin's solution for 1 h at 56 °C to improve the quality of the stain. Rinsed the sections thoroughly with running tap water for 5–10 min to remove any yellow color. Stained the sections with Weigert's iron hematoxylin solution for 10 min to color the nuclei. Rinsed the sections in running warm tap water for 10 min and then washed in distilled water. Stained the sections with Biebrich scarlet‐acid fuchsin solution for 10–15 min. Differentiated the sections in phosphomolybdic‐phosphotungstic acid solution for 10–15 min or until the collagen loses its red color. Transfered the sections directly to aniline blue solution and stained for 5–10 min. Rinsed briefly in distilled water and differentiated in 1% acetic acid solution for 2–5 min. Dehydrated the sections very quickly through 95% ethyl alcohol, absoluted ethyl alcohol, and cleared in xylene. Collagened fibers will be stained blue. Nuclei will be stained black. Muscle, cytoplasm, and keratin will be stained red. The background will be stained red.

Masson's trichrome‐stained kidney sections were scanned at 200× magnification. Five non‐overlapping cortical fields per animal were randomly selected and photographed. Using ImageJ software, the blue‐stained collagen area was segmented by color‐thresholding and normalized to the total parenchymal area. The fibrosis index was expressed as collagen volume fraction (CVF%, collagen area/total parenchymal area) ×100%. All analyses were performed blinded to group assignment by two independent investigators; inter‐observer variability was <5%. The mean value of the five fields was taken as the fibrosis score for each animal.^[^ ^59^ ^]^

### Immunohistochemistry (IHC) Staining

Paraffin‐embedded tissue sections were mounted on silane or poly‐L‐lysine coated slides. Deparaffinized the slides by incubating in a 60 °C oven for 30 min. Hydrated the slides through a series of xylene and graded ethanol washes, ending with distilled water. Placed the slides in a glass beaker containing 0.01 m citrate buffer, heated the solution until it boils, then maintained boiling for 10 min. Rinsed the slides with PBS. Incubated the slides in a peroxidase blocking solution for 10 min to quench endogenous peroxidase activity. Incubated the slides with a 10% normal goat serum for 1 hour at room temperature to reduce non‐specific binding. Applied the primary antibody α‐smooth muscle actin (α‐SMA) diluted in TBST with 1% BSA and incubated overnight at 4 °C. Washed the slides three times for 5 min each with TBST to remove unbound primary antibody. Applied the HRP‐conjugated secondary antibody diluted in TBST with 1% BSA and incubate for 1 hour at room temperature. Developed the colorimetric reaction using a DAB in the presence of HRP and hydrogen peroxide. Captured images using a microscope and analyzed the staining intensity and pattern.

### Immunofluorescence (IF) Staining

Brain and kidney tissue sections were performed as previously described. Immunofluorescence staining was performed as previously described. Paraffin sections were soaked in cold acetone for 15 min. After washing with phosphate buffered saline (PBS), pre‐cooled methanol was added dropwise and treated at −20 °C for 30 min. Sections were then blocked with 3% BSA for 60 min at room temperature. For co‐localization studies, primary antibodies against IBA‐1 were used in conjunction with antibodies against CD86, MDK, and P2ry12. Antibodies were added at room temperature and incubated overnight at 4 °C. Corresponding secondary antibodies were added for binding, followed by nuclei staining with DAPI. For kidney tissue sections, co‐localization of MDK with Fibronectin and α‐SMA was performed using the same protocol. For the detection of neuronal degeneration, Fluoro Jade B staining was carried out in accordance with the manufacturer's protocol.

Slides were visualized using a Nikon Eclipse Ti‐U fluorescence confocal microscope equipped with a digital camera. Co‐localization analysis was performed using Image J software. The software was used to quantify the overlap of fluorescent signals, indicating the co‐localization of the proteins of interest. Data obtained from Image J analysis were expressed as the mean ± standard error of the mean and were statistically analyzed using GraphPad Prism 6 or equivalent software.

### Morris Water Maze (MWM) Test

MWM test is a widely used behavioral assay for assessing spatial learning and memory in rodents.^[^ ^60^ ^]^ The maze is a circular pool, which is divided into four quadrants, with a hidden platform placed in the center of one quadrant, submerged 1–2 cm below the water surface. The water is made opaque with the addition of non‐toxic white tempera paint or by the use of a milky solution. A video tracking system is used to record the animal's path and measure latency to find the platform.

On the first day, a cued trial was conducted to ensure that animals can swim and locate a visible platform. The platform was raised above the water level with a flag or other prominent cue attached. Animals were released from each of the four quadrants and the latency to reach the platform was recorded. Animals were trained to find the hidden platform over a series of trials, typically 4 trials per day for 5–6 days. Each trial begins with the animal were placed in the water at one of the four start locations, facing the pool wall. The inter‐trial interval was kept between 15–30 s. If the animal did not find the platform within a set time limit, it was gently guided to the platform and allowed to remain there for 15 s. Latency to find the platform and the path taken were recorded using the video tracking system.

After the animal has reached asymptotic performance, a probe trial was conducted without the platform. The animal was released from a novel start position and the time spent in the target quadrant, as well as platform crossings, were recorded over a fixed interval. Statistical analysis of latency to find the platform, distance swum, and time spent in the target quadrant during probe trials were conducted to assess spatial learning and memory.

### Evans Blue (EB) Assay

The BBB is a critical interface that maintains brain homeostasis and protects the central nervous system from systemic influences.^[^ ^61^ ^]^ The EB assay is a widely used method for evaluating BBB integrity and permeability in rodent models.^[^ ^62^ ^]^ Weighed and anesthetized the mouse according to standard protocols, the mice were injected via the tail vein with 2% EB (4 mL kg^−1^). After 2 h circulation, the mice were euthanized and transcardially perfused with 0.9% NaCl until the outflow fluid from the right atrium was clear. Removed the brain and took pictures. Then, homogenized the brain in a saline solution (1 mL/g). EB was extracted from the homogenized tissue using formamide. Incubated the samples at 37 °C for 24 h to ensure complete extraction. Then, the supernatant was obtained by centrifugation of the tissue at 1000× *g* for 15 min. Finally, the quantity of extravasated EB in the sample was detected by a spectrophotometer at a wavelength of 632.

### Enzyme Linked Immunosorbent Assay (ELISA)

Collected blood from euthanized mice by cardiac puncture using a sterile syringe coated with an anticoagulant such as heparin to prevent clotting. Allowed the blood to coagulate at room temperature for 1–2 h. Centrifuged the blood at 1000–1500 g for 10–15 min to separate the serum. Transferred the serum to a clean tube and store at −20 °C or −80 °C until analysis.

The S100β protein is a calcium‐binding protein primarily expressed in the central nervous system and is involved in various physiological and pathological processes.^[^ ^63^ ^]^ Allowed samples to equilibrate to room temperature before use. Added 50 µL standard or sample to appropriate wells. Added 50 µL antibody cocktail to all wells. Incubated at room temperature for 1 h. Aspirated and washed each well three times with 1× Wash Buffer. Added 100 µL TMB Development Solution to each well and incubated for 5 min. Added 100 µL Stop Solution and readed the optical density (OD) at 450 nm. According to the manufacturer's instructions, detect the expression of MDK protein in mouse serum using a similar method as described above. Created a standard curve by plotting the average blank control subtracted absorbance value for each standard concentration against the target protein concentration and determined the concentration of the target protein in the sample.

### Flow Cytometry Analysis of Microglia

Euthanized mice and performed transcardiac perfusion with cold PBS to flush out blood. Removed the brain and mechanically dissect it into small pieces. Digested the tissue with enzymatic solutions for 30 min at 37 °C. Mechanically homogenized the tissue to obtain a single‐cell suspension. Used Percoll to separate myelin from cellular components. Centrifuged the samples to obtain a clean cell fraction. Incubated the cell suspension with the viability dye 7‐AAD to distinguish live cells. Labeled cells with fluorescently conjugated antibodies against CD11b, CD45, and other surface markers and analyzed the cells using a flow cytometer. Identified microglia as CD11b^+^CD45^int^ and activated microglia/macrophages as CD11b^+^CD45^high.[^ ^64^ ^]^ Used FlowJo to set gates and analyze the data, quantified the percentage and absolute numbers of microglia subsets.

### Western Blot Analysis

Tissues or cells were lysed using a RIPA buffer containing phosphatases and proteases inhibitors. Protein concentrations were determined using a BCA protein assay kit according to the manufacturer's instructions, ensuring equal amounts of protein were loaded per lane. Protein samples were resolved by SDS‐PAGE at a constant voltage. Proteins were transferred from the gel to a nitrocellulose or PVDF membrane using a wet transfer system. After transfer, membranes were blocked with 5% non‐fat milk in Tris‐buffered saline with Tween 20 for 1 h at room temperature to prevent non‐specific binding. Membranes were incubated with the primary antibody (Albumin, Occludin, MDK, Lrp1, P2ry12, and b‐Actin) specific to the target protein overnight at 4 °C. After washing with TBST, membranes were incubated with horseradish peroxidase‐conjugated secondary antibodies for 1 hour at room temperature. The antibody information used in the experiment is shown in Supplementary Table S1 (Supporting Information). Protein bands were visualized using an enhanced chemiluminescence detection system and imaged using a ChemiDoc imaging system. Densitometry analysis of the bands was performed using Image Lab software to quantify the protein expression levels relative to the loading control.

### Quantitative Real‐Time Polymerase Chain Reaction (qRT‐PCR)

Total RNA was extracted from cells using the RNeasy Mini Kit according to the manufacturer's protocol. The quality and concentration of the extracted RNA were assessed using a NanoDrop spectrophotometer. Reverse transcription was performed using the High‐Capacity cDNA Reverse Transcription Kit with 1 µg of total RNA per reaction.

The qRT‐PCR was performed using a SYBR Green Master Mix on a StepOnePlus Real‐Time PCR System. Each reaction consisted of 2× qPCR Mix, 2 µM of each primer, and diluted cDNA template in a total volume of 15 µL. The amplification program included an initial denaturation at 95 °C for 30 s, followed by 40 cycles of 95 °C for 10 s, and 60 °C for 20 s. A melting curve analysis was performed at the end of each run to confirm the specificity of the amplification. The relative expression levels of target genes were calculated using the 2^−ΔΔCt^ method, where ΔCt was the difference between the Ct values of the GAPDH. The ΔΔCt was the difference between the ΔCt of the treated samples and the average ΔCt of the control samples, which allowed for the calculation of the fold change in gene expression relative to the control. Data were analyzed using staphPad, and the gene sequence is shown in Supplementary Table S2 (Supporting Information).

### Cell Culture and Plasmid Transfection

HK2 (human renal proximal tubular epithelial cells), NRK‐52e (normal rat kidney epithelial cells), and NRK‐49F (normal rat kidney fibroblasts) cells were cultured in DMEM medium or DMEM/F12 medium, 10% (v/v) fetal bovine serum (FBS), and 1% (v/v) penicillin/streptomycin. The cells were maintained at 37 °C in a humidified atmosphere containing 5% CO2. BV2 cultures were passaged at a split ratio of 1:2 to 1:4, and media was renewed 2 to 3 times per week.

For the overexpression of MDK, the full‐length MDK cDNA was cloned into a mammalian expression vector under the control of a CMV promoter. HK2, NRK‐52E, and NRK‐49F cells were transfected with the MDK overexpression plasmid or control vectors using a standard calcium phosphate transfection method. Briefly, cells were seeded at a density of 2 × 10^5^ cells per well in a 6‐well plate and allowed to reach 70–80% confluence. The transfection mixture was prepared by mixing 2 µg of plasmid DNA with an equal volume of 2× HEPES‐buffered saline (HBS) and adding this to an equal volume of 2× calcium phosphate solution. The mixture was incubated at room temperature for 20 min before being added dropwise to the cells. After a 4‐hour incubation at 37 °C, the medium was replaced with fresh complete medium. The efficiency of MDK overexpression was evaluated using qRT‐PCR and western blot analysis.

### Lrp1 Small Interfering RNA (siRNA) Transfection

Lrp1 was silenced with Lrp1‐siRNA, and equal amounts of non‐specific sham siRNA (NC) were used for control. Primary microglia were transfected with 40 nM (final concentration) of LipoRNAi Transfection Reagent. After 48 h, the efficiency of siRNA‐mediated gene silencing was confirmed by qRT‐PCR. Recombinant MDK (100 ng mL^−1^) was added after 24 h of siRNA. The cells were collected after 24 h to detected the expression of MDK by western blotting. The sequence of Lrp1‐siRNA1 is shown in Table S3 (Supporting Information).

### Co‐Culture System

MDK‐overexpressing HK2 (MDK^hi^‐HK2), 52E (MDK^hi^‐52E) and NRK49F (MDK^hi^‐NRK49F) cells were co‐cultured with BV2 microglia or Lrp1^−/−^ microglia using a transwell system. This system allowed for the separation of the two cell types while allowing for soluble factors to pass through and influence each other. The transwell inserted with a pore size of 0.4 µm were used to ensure that only small molecules and not cells could pass between the compartments. The expression levels of MDK, Lrp1 and P2ry12 and inflammatory factors were evaluated using qRT‐PCR and western blot analysis.

### Phagocytosis Assay

The phagocytic function of primary microglia was assessed using a fluorescent bead assay.^[^ ^64^ ^]^ Fluorescently labeled 1‐µm polystyrene beads were mixed with FBS and incubated with the cells at a bead‐to‐cell ratio of 10:1 for 4 h at 37 °C. After incubation, cells were gently washed with PBS to remove non‐ingested beads, and then fixed with 4% paraformaldehyde for 15 min at room temperature. The number of ingested beads was quantified using flow cytometry by measuring the mean fluorescence intensity of the cells.

Flow cytometry data were analyzed using FlowJo software or equivalent. Gates were set based on unstained and single bead controls to exclude debris and accurately count phagocytic events.

### Single‐Cell Preparation for RNA Sequencing

Kidney and hippocampal tissues were minced into small pieces (<1 mm^3^) and incubated in tissue dissociation buffer [Liberase TM (1 mg mL^−1^), hyaluronidase (0.7 mg mL^−1^), and deoxyribonuclease (80 U/mL) in PBS] for 30 min at 37 °C. Single cells were released from the digested tissue by pipetting 10 times, and the cell suspension was filtered through a 70‐µm sieve (Falcon). Fetal bovine serum (10%) was added to stop the enzymatic reaction. Cells were collected by centrifugation (300 g at 4 °C for 5 min) and resuspended in red blood cell (RBC) lysis buffer for 1 min at room temperature. After washing in PBS, cells were used fresh for analysis by scRNA‐seq.

### Single‐Cell RNA Sequencing (scRNA‐seq)

Cells were stained with propidium iodide, and live cells were sorted using FACSAria III. Libraries were prepared using the Chromium Single Cell 5′ Library Kit v2 and Chromium instrument. Full‐length cDNA was amplified, and libraries were submitted to Genome Technology Access Center of Washington University in St. Louis for sequencing at a depth of 50 000 reads. All processing steps were performed using Seurat v3. Quality control was performed on each library to find appropriate filtering thresholds. Expression matrices for each sample were loaded into R as Seurat objects, retaining only cells with more than 200 and less than 3200 genes. Poor quality cells with >10% mitochondrial genes were removed. Any gene not expressed in at least three cells was removed. Sc‐transform was used for normalization scaling and variance stabilization. Integration of kidney and hippocampal single‐cell data was done using the harmony package to control for batch effects. After quality control and integration, cells were further analyzed. We identified clusters in the kidney and hippocampus and visualized cell clustering using UMAP. To assign cluster identities, a list of kidney and hippocampal cell types and their established markers was compiled, and the expression of those markers was assessed using the FindAllMarkers() function in Seurat. scRNA‐seq mentioned above was performed by Shanghai Gene Chemical Co., Ltd.

### Ligand‐Recepto (L‐R) Pairing Analysis

CellChat was used to infer cell‐cell communication by analyzing the expression of L‐R pairs between kidney and hippocampus cell populations. CellChat contains a highly curated set of human protein‐protein interactions and protein complexes. All datasets from kidney (Sham, IRI‐7, IRI‐28) and hippocampus (Sham, IRI‐7, IRI‐28) were integrated using reciprocal principal components analysis as implemented in Seurat. The analysis was performed with default settings, considering only those L‐R pairs where the ligand was expressed in kidney cells and the receptor was expressed in hippocampal cells. Mouse genes were mapped to their high‐confidence human one‐to‐one orthologs using homology mappings from Ensemble. CellChat statistical analysis was performed with default settings between all kidney and hippocampal cell populations to increase statistical power. Last, we considered only co‐expressed pairs with a ligand expressed in a kidney cell population and its cognate receptor expressed in a hippocampus cell population with significant cell type–specific co‐expression as compared to randomly shuffled cells (*p* < 0.01), with higher P values indicating higher significance.

### Statistical Analysis

For the animal and cell culture sample studies, values were presented as the mean ± standard error of the mean (SEM). For in vitro studies, each experiment was performed independently at least three times with distinct cell preparations. For in vivo studies, the exact sample size (n) for each group was provided in the figure legends. All animal experiments were repeated independently at least twice with similar outcomes. Statistical analyses were carried out using GraphPad Prism 7. Comparisons between two means were made by Student's t test; comparisons between three or more means were made by one‐way analysis of variance (ANOVA) with Tukey's multiple comparisons tests. Correlation analysis was performed using Spearman's correlation test. A *p* value < 0.05 was considered statistically significant.

## Conflict of Interest

The authors declare no conflict of interest.

## Author Contributions

L.L., B.L., and Y.Y. contributed equally to this work and share first authorship. L.L. performed animal surgeries, most of the tissue analysis, protein assays and designed figures, performed flow cytometry experiments, supervised experiments and data analysis, performed the manuscript writing and editing, and provided the funding support; L.B. performed the scRNAseq analysis, contributed to tissue analysis, carried out the CellChat analysis and provided help with the scRNAseq analysis; Y.Y. provided expertise for analysis of the kidney phenotype, helped with animal surgeries and BUN ELISA; M.T. helped with design, technical aspects, and analysis of scRNAseq experiments; C.Y. performed mouse MDK and Albumin ELISAs, as well as part of the qPCR measurements; P.Y. and G.J. assisted in partial Western blot and PCR detection; G.H. provided a platform and resources for experiments; L.Z. revised the manuscript; Z.X. conceived the idea for the project, designed all experiments, supervised their analysis, and provided platform and funding support.

## Supporting information



Supporting Information

## Data Availability

The data that support the findings of this study are available on request from the corresponding author. The data are not publicly available due to privacy or ethical restrictions.
