# Supplementary material for: Midkine‐Mediated Microglia Activation after Renal Injury Promotes Cognitive Impairment Following Ischemic Renal Injury
Source: Adv Sci (Weinh). 2025 Nov 23;13(3):e07832. doi: 10.1002/advs.202507832 (PMC12806390; doi:10.1002/advs.202507832)
Supplement: Supplementary file 1 — Supporting Information [file ADVS-13-e07832-s001.docx]

**Midkine-Mediated Microglia Activation After Renal Injury Promotes Cognitive Impairment Following Ischemic Renal Injury**

Li Lu^1,2#^, Bixiao Liu^1#^, Yu Yang^1#^, Tao Meng^1^, Yuanyuan Chang^3^, Yao Peng^1^, Jia Guo^1^, Zhuqing Wang^1^, Hongqian Guo^2^*, Liuhua Zhou^1^*, Xiaozhi Zhao^1,2^*

# These authors have contributed equally to this work and share first authorship.

**This file includes:**

**Table. S1-S5**

**Figures. S1 to S7**

**Supplementary Table 1: experimental materials**

| **REAGENT or RESOURCE** | **SOURCE** | **IDENTIFIER** |
| --- | --- | --- |
| **Flow cytometry antibody** | | |
| **CD45 Monoclonal Antibody (30-F11), FITC** | **Abcam** | **Cat# ab210225** |
| **CD11b monoclonal [M1/70], APC** | **Abcam** | **Cat# ab25482** |
| **CD86 Monoclonal Antibody (GL1), PE-Cyanine7** | **Thermo Fisher Scientific** | **Cat# A15412** |
| **CD206 Monoclonal Antibody (MR5D3), PE** | **Thermo Fisher Scientific** | **Cat# MA5-16872** |
| **WB and IF antibody** | | |
| **Anti-Beta Actin antibody [2D4H5]** | **Proteintech** | **Cat# 66009-1-Ig** |
| **Anti-Albumin antibody [P07724]** | **Bioworld** | **Cat# BS90045** |
| **Anti-Occludin antibody [E6B4R]** | **Cell Signaling Technology** | **Cat# 91131S** |
| **Anti-MDK antibody [P21741]** | **Bioworld** | **Cat# BS6038** |
| **Anti-Fibronectin antibody [1G10F9]** | **Proteintech** | **Cat# 66042-1-Ig** |
| **Anti-a-SMA antibody [1E9A11]** | **Proteintech** | **Cat# 67735-1-Ig** |
| **Anti-Iba-1 antibody [EPR16588]** | **Abcam** | **Cat# ab178846** |
| **Anti-NeuN antibody [EPR12763]** | **Abcam** | **Cat# ab177487** |
| **Anti-Flag antibody [9A3]** | **Cell Signaling Technology** | **Cat# 8146S** |
| **Anti-Lrp1 antibody [EPR3724]** | **Abcam** | **Cat# ab92544** |
| **Anti-P2ry12 antibody [Q9H244]** | **Proteintech** | **Cat# 11976-1-AP** |
| **Anti-rabbit IgG-HRP antibody** | **Santa Cruz Biotechnology** | **Cat# sc-2077** |
| **Anti-mouse IgG-HRP antibody** | **Santa Cruz Biotechnology** | **Cat# sc-2314** |
| **Anti-Rabbit IgG, Alexa Fluor 488**  **conjugated** | **Thermo Fisher Scientific** | **Cat# A-11034** |
| **Anti-Mouse IgG, Alexa Fluor 488**  **conjugated** | **Thermo Fisher Scientific** | **Cat# R37120** |
| **Anti-Mouse IgG, Alexa Fluor 594**  **conjugated** | **Cell Signaling Technology** | **Cat# 8890S** |
| **Anti-Rabbit IgG, Alexa Fluor 594**  **conjugated** | **Cell Signaling Technology** | **Cat# 8889S** |
| **Elisa Kit** | | |
| **TUNEL Cell Apoptosis Detection Kit (Green Fluorescence)** | **Beyotime** | **Cat# C1086** |
| **S100β ELISA kit** | **Abcam** | **Cat# ab234573** |
| **MDK ELISA kit** | **Abcam** | **Cat# ab279416** |
| **Masson trichrome staining kit** | **Beyotime** | **Cat# C0189S** |
| **Chemicals, peptides, and recombinant proteins** | | |
| **Triton X-100** | **Sigma** | **Cat# X100PC** |
| **DAPI** | **Beyotime** | **Cat# C1002** |
| **RPMI 1640** | **Gibco** | **Cat# 12633012** |
| **DMEM** | **Gibco** | **Cat# 11965092** |
| **DMEM/F-12** | **Gibco** | **Cat# 11320082** |
| **TRIzol RNA isolation reagent** | **Beyotime** | **Cat# R0016** |
| **ChamQ SYBR qPCR Master Mix** | **Vazyme** | **Cat# Q311-02/03** |
| **RIPA buffer** | **Beyotime** | **Cat# P0013C** |
| **BCA Protein Assay Kit II** | **Abcam** | **Cat# ab287853** |
| **ECL chemiluminescence detection kit** | **Vazyme** | **Cat# E411-04/05** |
| **Biostep™ Pre stained protein marker** | **Tanon** | **Cat# 180-6006** |
| **PVDF Transfer Membranes** | **Thermo Fisher Scientific** | **Cat# 88520** |
| **One-Step PAGE Gel Fast Preparation Kit** | **Vazyme** | **Cat# E303-01** |
| **Percoll®** | **Sigma** | **Cat# P1644** |
| **MDK/Midkine Protein, Mouse (His)** | **MCE** | **Cat# HY-P701025** |

**Supplementary Table 2: Primers used for Real-time PCR**

| **Gene** | **sense（5'-3'）** | **antisense（5'-3'）** |
| --- | --- | --- |
| ***m-Actb*** | TCCGGCACTACCGAGTTATC | GATCCGGTGTAGCAGATCGC |
| ***m-IL-1a*** | GCACCTTACACCTACCAGAGT | AAACTTCTGCCTGACGAGCTT |
| ***m-IL-1b*** | GCAACTGTTCCTGAACTCAACT | ATCTTTTGGGGTCCGTCAACT |
| ***m-IL-2*** | GTGCTCCTTGTCAACAGCG | GGGGAGTTTCAGGTTCCTGTA |
| ***m-IL-6*** | TAGTCCTTCCTACCCCAATTTCC | TTGGTCCTTAGCCACTCCTTC |
| ***m-IL-10*** | GCTCTTACTGACTGGCATGAG | CGCAGCTCTAGGAGCATGTG |
| ***m-IL-12*** | AGAGAATGCTCATTGGCACTTC | AACTGGGATAATGTGAACAGCC |
| ***m-IFN-γ*** | ATGAACGCTACACACTGCATC | CCATCCTTTTGCCAGTTCCTC |
| ***m-TNF-a*** | CCCTCACACTCAGATCATCTTCT | GCTACGACGTGGGCTACAG |
| ***m-CCL2*** | TTAAAAACCTGGATCGGAACCAA | GCATTAGCTTCAGATTTACGGGT |
| ***m-Midkine*** | GAAGAAGGCGCGGTACAATG | GAGGTGCAGGGCTTAGTCA |
| ***h-Midkine*** | GAAGAAGGCGCGGTACAATG | GAGGTGCAGGGCTTAGTCA |
| ***m-Trem2*** | CTGGAACCGTCACCATCACTC | CGAAACTCGATGACTCCTCGG |
| ***m-Cx3cr1*** | GAGTATGACGATTCTGCTGAGG | CAGACCGAACGTGAAGACGAG |
| ***m-Tmem119*** | CCTACTCTGTGTCACTCCCG | CACGTACTGCCGGAAGAAATC |
| ***m-CD200R*** | CCAAGTGCCAGGGGAAAATAG | TTGCTCCTGACAATCATGGTG |
| ***m-P2ry12*** | CCCTGTGCGTCAGAGACTAC | CAAGCTGTTCGTGATGAGCC |
| ***m-Lrp1*** | ACTATGGATGCCCCTAAAACTTG | GCAATCTCTTTCACCGTCACA |
| ***m-ItgA6*** | TGCAGAGGGCGAACAGAAC | GCACACGTCACCACTTTGC |
| ***m-Itgb1*** | TGTGGGCAACACTTTGACCC | CACAGTACAGCCCTTGATGTTTA |

**Supplementary Table 3: Lrp1-siRNA1 sequence**

| **Gene** | **sense（5'-3'）** | **antisense（5'-3'）** |
| --- | --- | --- |
| ***Lrp1-siRNA1*** | GGGCCAUGAAUGUGGAAAUTT | AUUUCCACAUUCAUGGCCCTT |
| ***Lrp1-siRNA2*** | GCCCAUUGGAUGAGUUUCATT | UGAAACUCAUCCAAUGGGCTT |
| ***Lrp1-siRNA3*** | GGUGCCUGAUAUCGACAAUTT | AUUGUCGAUAUCAGGCACCTT |
| ***Lrp1-siRNA4*** | CGGGAAACUUCUACUUUGUTT | ACAAAGUAGAAGUUUCCCGTT |
| ***ItgA6-siRNA1*** | CGGAAAUCCUUUCAAGAGAAATT | UUUCUCUUGAAAGGAUUUCCGTT |
| ***ItgA6-siRNA2*** | CCAGGGACUUACAACUGGAAATT | UUUCCAGUUGUAAGUCCCUGGTT |
| ***Itgb1-siRNA1*** | CAACGCAUAUCUGGAAACUUGGAUU | AAUCCAAGUUUCCAGAUAUGCGUUG |
| ***Itgb1-siRNA2*** | GAGGUCGUUCUUCAGUUCAUCUGUA | UACAGAUGAACUGAAGAACGACCUC |
| ***Itgb1-siRNA3*** | GCGUGGUUGCUGGAAUUGUUCUUAU | AUAAGAACAAUUCCAGCAACCACGC |

**Supplemental Figure 4: Software and algorithms**

| **REAGENT or RESOURCE** | **SOURCE** | **IDENTIFIER** |
| --- | --- | --- |
| **GraphPad Prism 8** | **Graphpad** | **NA** |
| **Fiji-ImageJ** | **National Inst. Of Health** | **NA** |
| **Bio-Rad Chemidoc XRS Gel Imaging**  **System** | **Bio-Rad** | **NA** |
| **FlowJo™ Software v10.10** | **BD** | **NA** |

**Supplemental Figure 5: Abbreviation**

| **abbreviation** | **full name** |
| --- | --- |
| AKI | Acute kidney injury |
| α-SMA | α-smooth muscle actin |
| BBB | Blood-brain barrier |
| BUN | Blood urea nitrogen |
| CKD | Chronic kidney disease |
| DTL-ATL | Descending thin limb and ascending thin limb |
| EB | Evans Blue |
| ELISA | Enzyme Linked Immunosorbent Assay |
| FBS | Fetal bovine serum |
| H&E | Hematoxylin and Eosin |
| IF | Immunofluorescence |
| IHC | Immunohistochemistry |
| IRI | Ischemia-reperfusion injury |
| L-R | Ligand-receptor |
| LRP1 | Lipoprotein Receptor-Related Protein 1 |
| MDK | Midkine |
| MWM | Morris Water Maze |
| OD | Optical density |
| PBS | Phosphate buffered saline |
| PFA | Paraformaldehyde |
| PT | Proximal tubules |
| qRT-PCR | Quantitative Real-Time Polymerase Chain Reaction |
| RBC | Red blood cell |
| SEM | Standard error of the mean |
| siRNA | Small interfering RNA |
| snRNA-seq | Single-nucleus RNA sequencing |
| WB | Western Blot |

**Supplementary Figure 1**


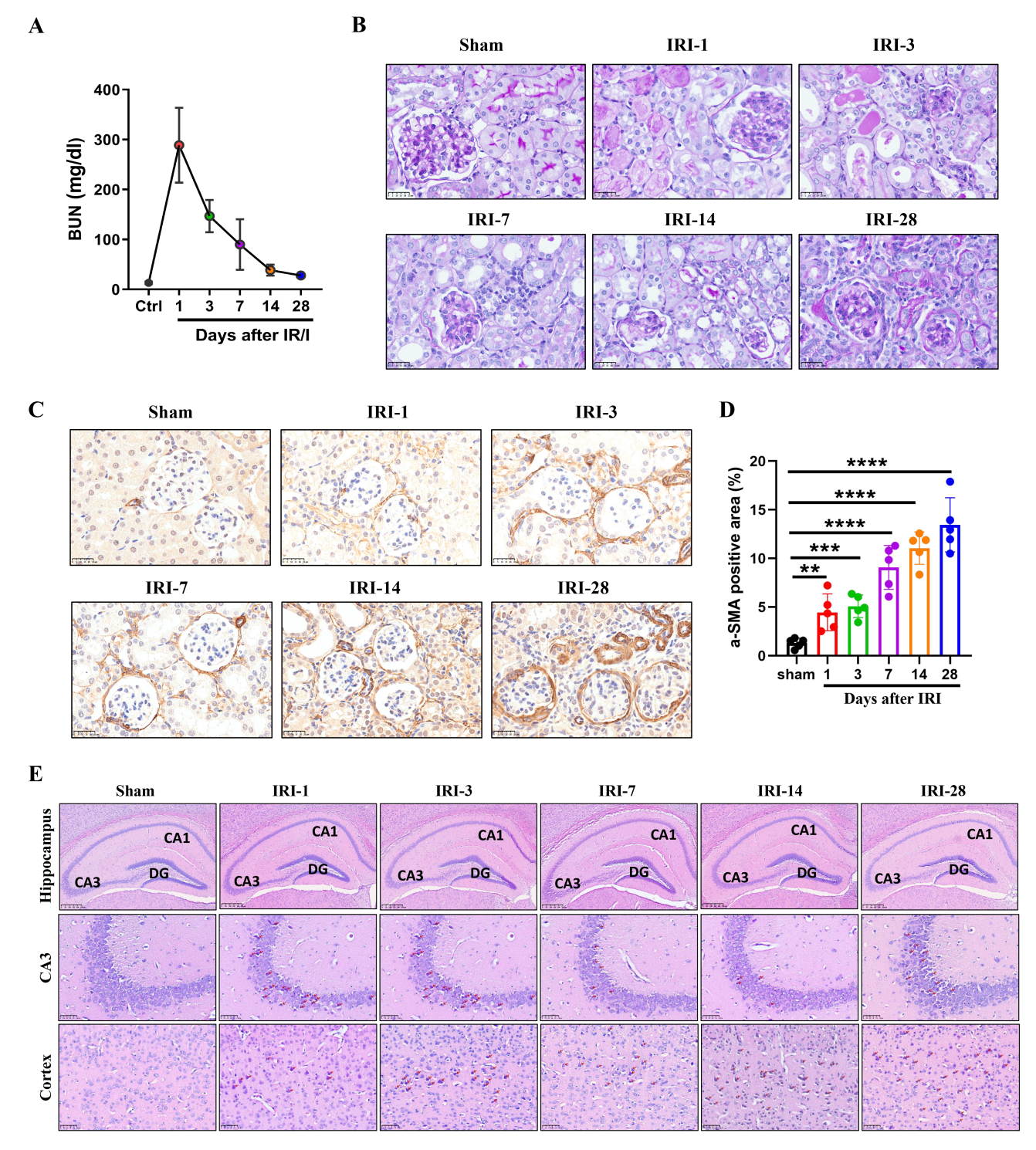


**Supplementary Figure 1:**

**(A).** Serum BUN levels were measured in mice. n=5

**(B).** PAS staining was performed on kidney tissues to visualize the renal structure, with a scale bar of 25 μm for reference.

**(C).** Immunohistochemical staining for α-SMA in mouse kidneys was conducted to assess fibrosis, scale bar represents 25 μm.

**(D).** Quantitative analysis of α-SMA staining intensity was conducted to evaluate the extent of fibrosis. n=5

**(E).** H&E staining of brain tissue sections was performed to assess the structural integrity of hippocampal regions, including CA3 and DG. Scale bars represent 250 μm for hippocampal tissue and 100 μm for CA3 and Cortex regions.

Data are presented as mean ± SEM, with statistical significance indicated by asterisks (**p < 0.01, ***p < 0.001, ****p < 0.0001).

**Supplemental Figure 2**

**
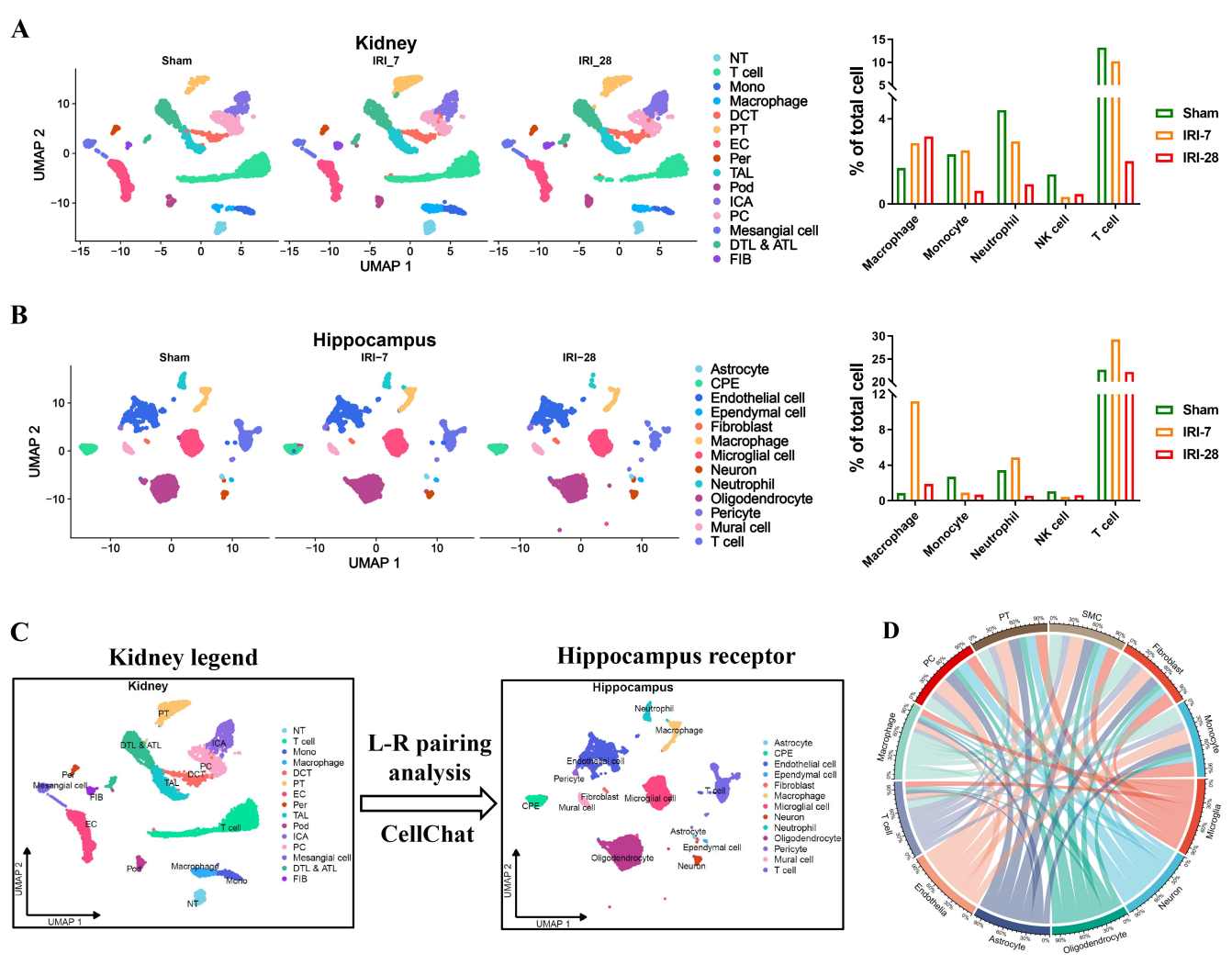
**

**Supplementary Figure 2:**

**(A).** Seurat objects: sham, IRI-7 and IRI-28 kidney, quantification of Macrophage, Monocytes, Neutrophils, NK cells and T cells in above three group expressed as the percentage of total cells detected by Seurat.

**(B).** Seurat objects: sham, IRI-7 and IRI-28 hippocampus, quantification of Macrophage, Monocytes, Neutrophils, NK cells and T cells in above three group expressed as the percentage of total cells detected by Seurat.

**(C).** Design of L-R pairing analysis, kidney ligands paired to Hippocampus receptors using kidney and hippocampus scRNA-seq data and CellChat analysis.

**(D).** L-R pairing analysis kidney cells to Hippocampus nonimmune cells . Cell epithelial: NT, neutrophil; Mono, Monocyte; DCT, Distal convoluted tubule; PT, proximal tubule; EC, endothelial cell; Per, pericyte; TAL, thick ascending limb; Pod, Podocytes; ICA, Collecting duct-Intercalated cell; PC, Principle cells; DTL&ATL, Decending thin/Thin ascending limb; Fib, Fibroblasts.

**Supplemental Figure 3**


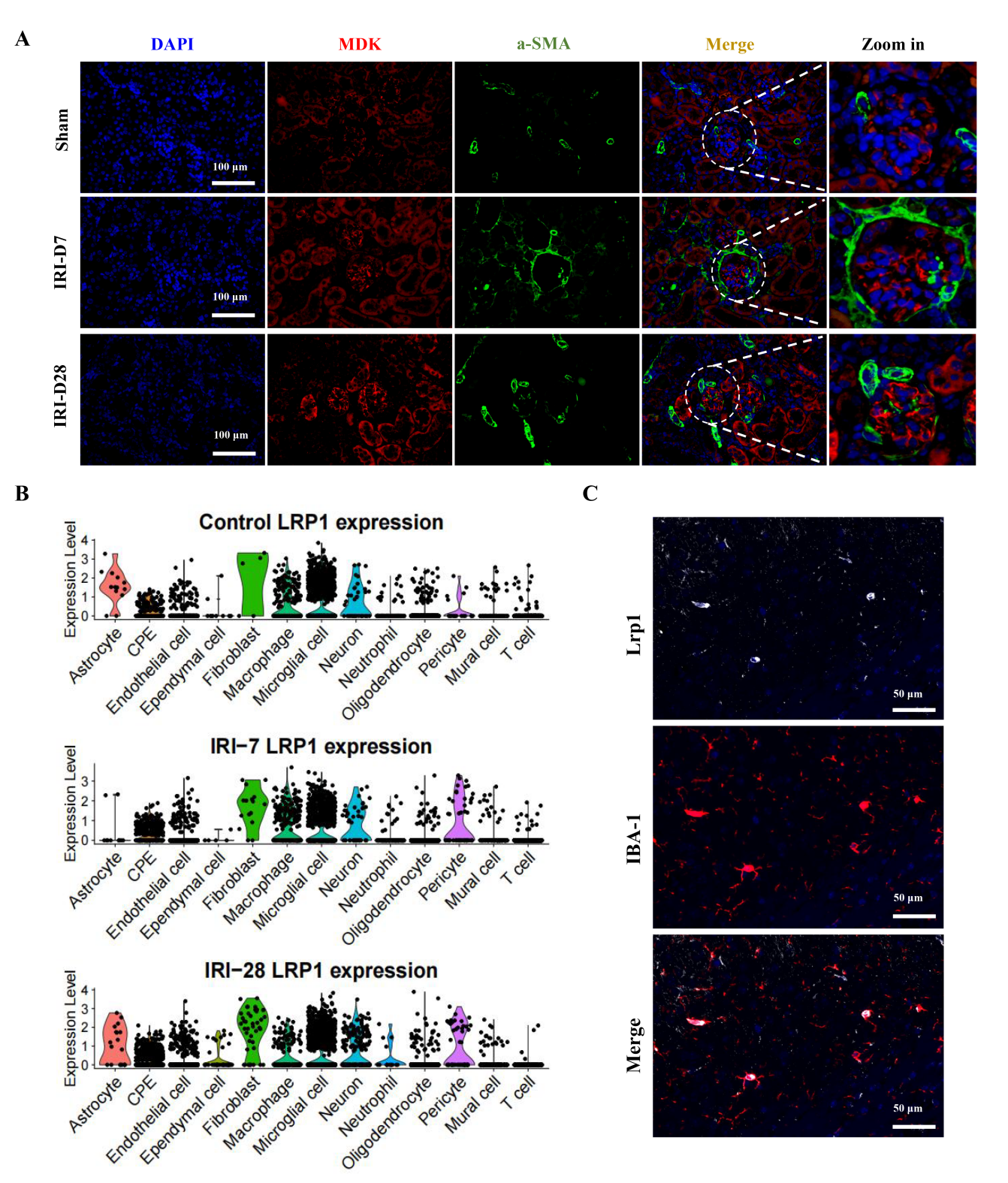


**Supplemental Figure 3:**

**(A).** Immunofluorescence analysis of kidney sections showing the expression of α-SMA and MDK in Sham, IRI-7, and IRI-28 groups. DAPI (blue) was used to stain nuclei. The merged images illustrate the co-localization of MDK and α-SMA (green and red, respectively), with a zoom-in inset highlighting the areas of interest. Scale bar represents 50 μm.

**(B).** Analysis of LRP1 expression across various cell types in the kidney by sc-RNA-seq analyzing, including Sham and IRI-7 and IRI-28 conditions.

**(C).** Immunofluorescence staining for LRP1 and IBA-1 in hippocampus sections. The merged images show the co-localization of LRP1 (white) and IB-1 (red), with DAPI (blue) staining nuclei. Scale bar represents 50 μm.

**Supplemental Figure 4**


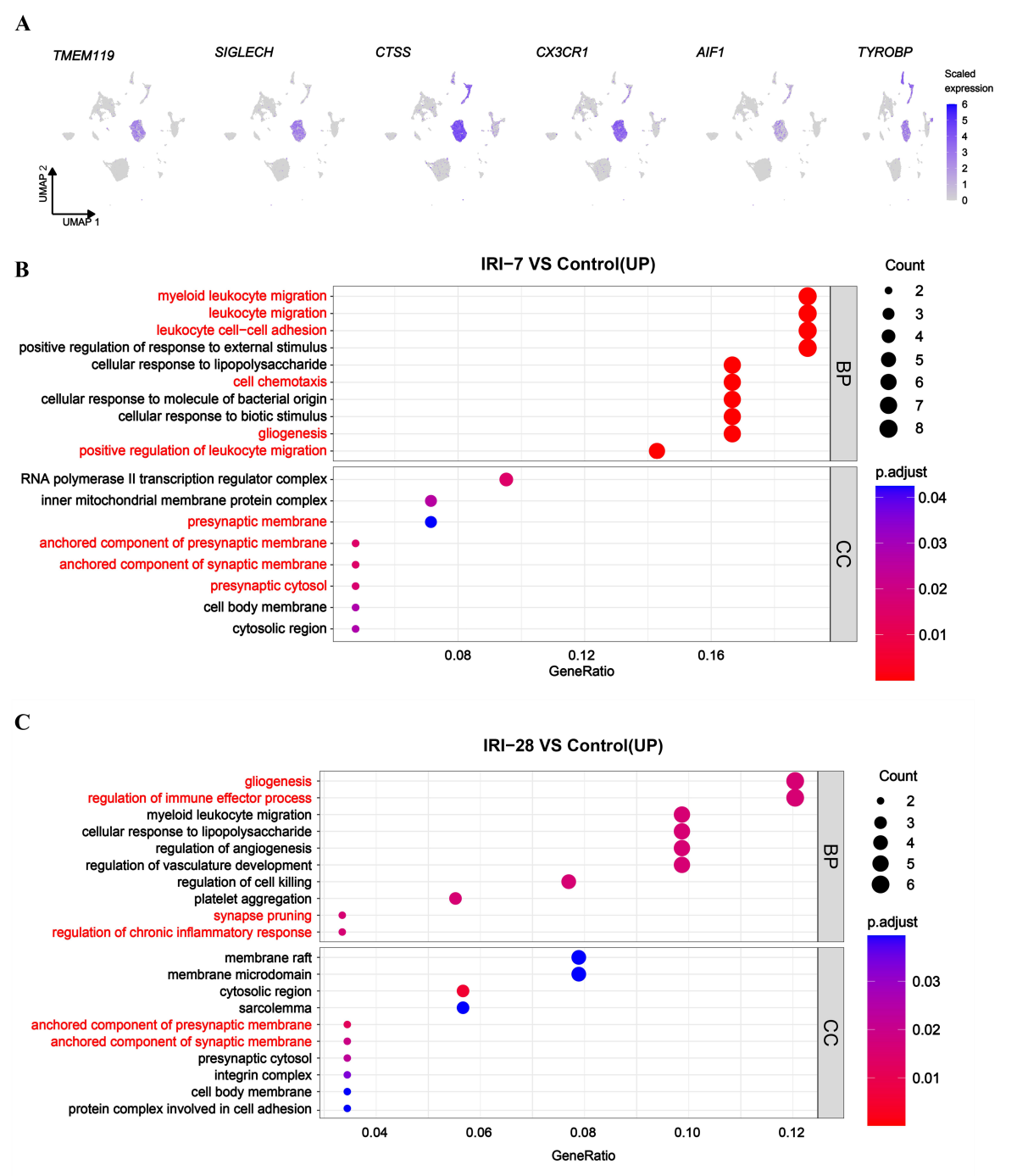


**Supplemental Figure 4:**

1. **.** Expression of indicated genes in microglia cells.
2. **-(C).** GO enrichment analysis for upregulated genes in the IRI-7 and IRI-28 compared to the control group. The scatter plot displays the GeneRatio (x-axis), which represents the proportion of DEGs in a specific GO category relative to the total number of genes in that category, against the Benjamini-Hochberg adjusted p-value (BP, y-axis). Larger dots indicate a higher count of genes within the category. The color gradient from blue to red corresponds to the p.adjust values, with red representing more significant enrichment (p.adjust < 0.01).

**Supplemental Figure 5**


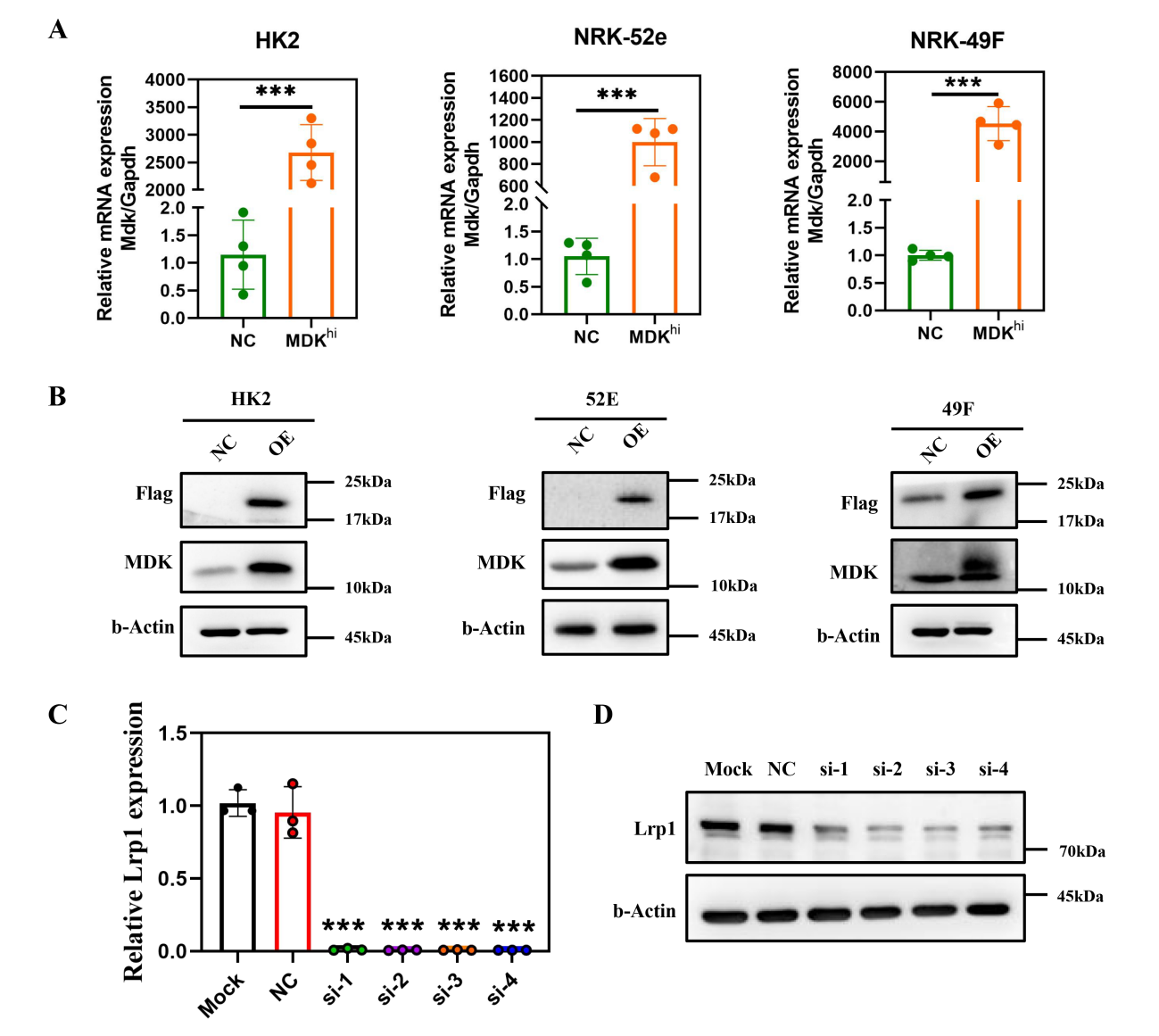


**Supplemental Figure 5:**

**(A).** Bar graphs representing the relative mRNA expression levels of MDK in HK2, NRK-52e, and NRK-49F cells transfected with MDK compared to NC cells.

**(B).** Western blot analysis of MDK protein expression transfected with MDK (Flag-tagged) or NC. The blots show the expression of the target proteins (Flag and MDK) and β-actin as a loading control.

**(C).** The bar chart represents the relative mRNA expression levels of Lrp1 in BV2 cells transfected with four types of siRNA targeting Lrp1 (si-1, si-2, si-3 and si-4) compared to NC.

**(D).** Western blot analysis of Lrp1 protein expression transfected with si-Lrp1 or NC. Data are presented as mean ± SEM, with statistical significance indicated by asterisks (***p < 0.001).

**Supplemental Figure 6**


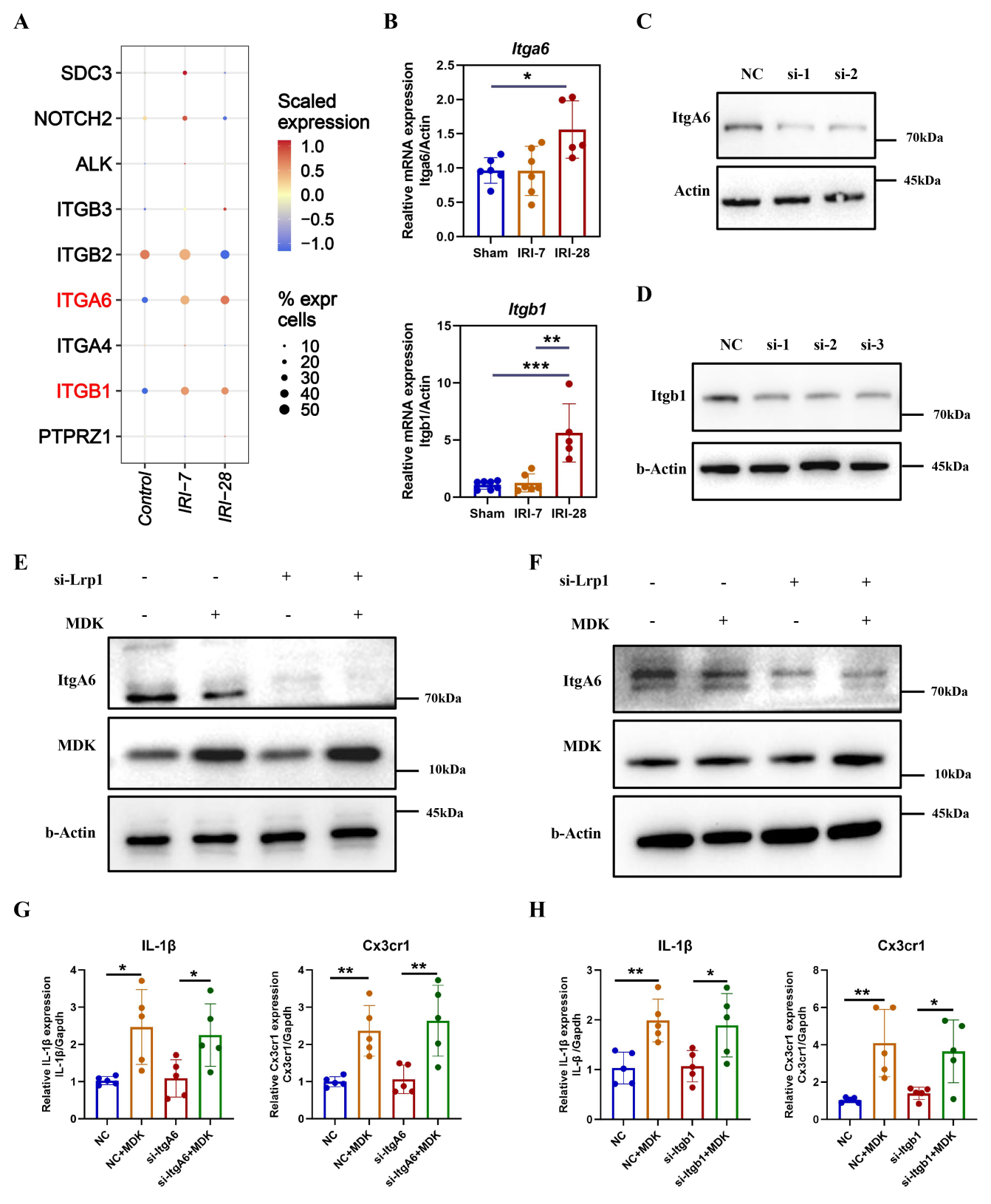


**Supplemental Figure 6:**

**(A).** The bar chart represents the relative mRNA expression levels of Itga6 and Itgb1 in BV2 cells transfected with four types of siRNA targeting Itga6 and Itgb1 (si-1, si-2 and si-3) compared to NC.

**(B).** Western blot analysis of Itga6 and Itgb1 protein expression transfected with si-Lrp1 or NC.

**(C).** Western blot analysis of ItgA6 protein expression transfected with si-ItgA6 or NC.

**(D).** Western blot analysis of Itgb1 protein expression transfected with si-Itgb1 or NC.

**(E).** Western blot analysis of ItgA6 and MDK expression in BV2 cells transfected with si-ItgA6 and treated with rMDK. β-Actin is used as a loading control.

**(F).** Western blot analysis of Itgb1 and MDK expression in BV2 cells transfected with si-Itgb1 and treated with rMDK. β-Actin is used as a loading control.

**(G)**. Bar graphs showing the relative mRNA expression levels of inflammatory cytokines in BV2 cells transfected with si-ItgA6 or si-NC and treated with rMDK.

**(H)**. Bar graphs showing the relative mRNA expression levels of inflammatory cytokines in BV2 cells transfected with si-Itgb1 or si-NC and treated with rMDK.

Data are presented as mean ± SEM, with statistical significance indicated by asterisks (***p < 0.001).

**Supplemental Figure 7**

**
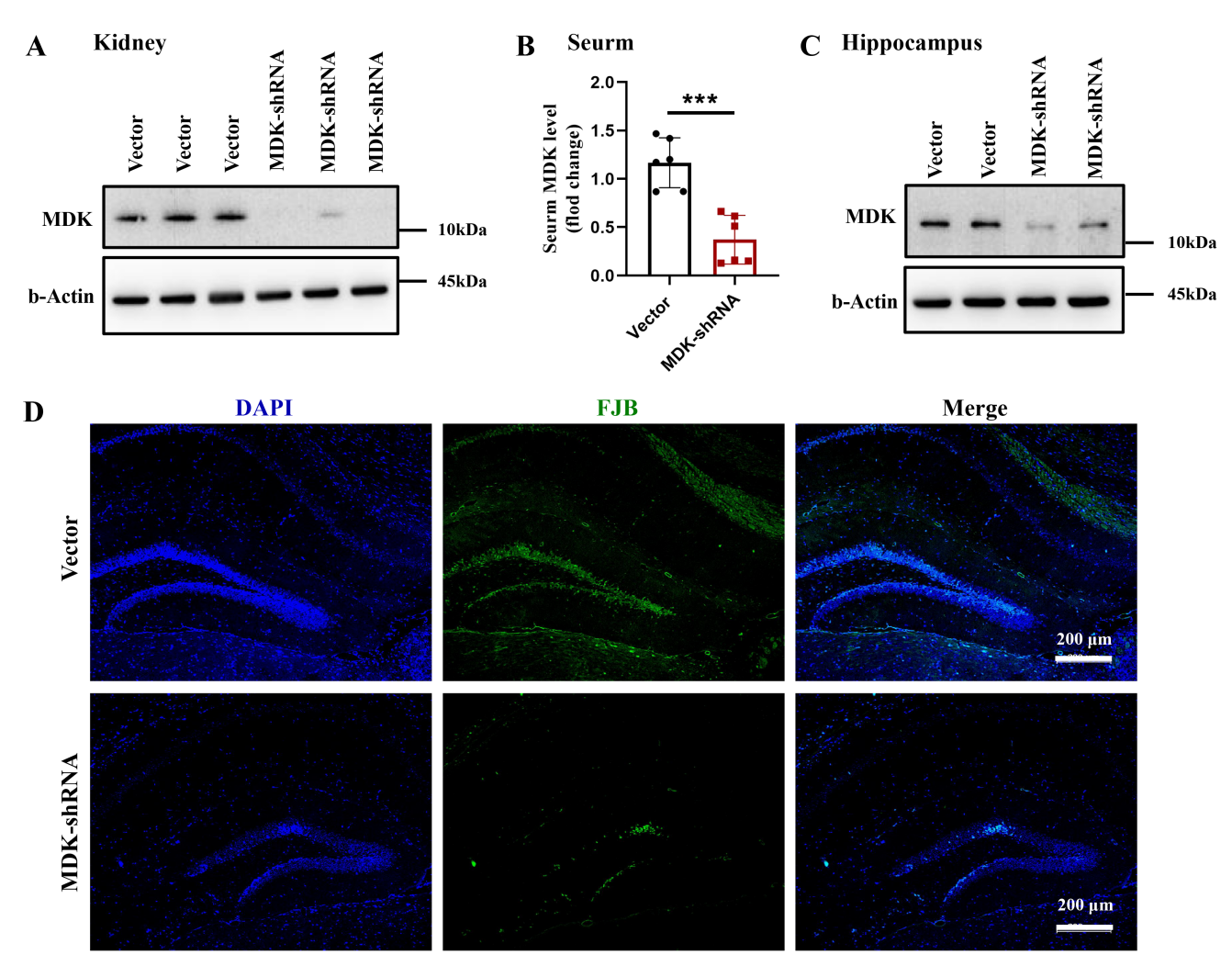
**

**Supplemental Figure 7:**

**(A).** Western blot analysis of MDK expression in kidney at vector group and MDK-shRNA group.

**(B).** Quantification of relative expression of MDK protein in serum at vector group and MDK-shRNA group.

**(C).** Western blot analysis of MDK expression in hippocampus at vector group and MDK-shRNA group.

**(D).** Representative fluorescent images of hippocampal FJB staining of mice, scale bar is 200 μm; and the number of FJB positive cells in the entire hippocampus.

Data are presented as mean ± SEM, with statistical significance indicated by asterisks (***p < 0.001).
